# Supplementary material for: Effect of Pennisetum giganteum z.x.lin mixed nitrogen-fixing bacterial fertilizer on the growth, quality, soil fertility and bacterial community of pakchoi (Brassica chinensis L.)
Source: PLoS One. 2020 Feb 12;15(2):e0228709. doi: 10.1371/journal.pone.0228709 (PMC7015373; doi:10.1371/journal.pone.0228709)
Supplement: S1 File — (DOCX) [file pone.0228709.s001.docx]

**Table 4. Analysis of soil bacterial community diversity under different fertilization treatments.**

| Sample ID | chao1 | goods_coverage | shannon |
| --- | --- | --- | --- |
| CK_1 | 3505.768671416 | 0.934224883566 | 9.401829385949 |
| CK_2 | 4137.582973533 | 0.9232601463741 | 9.850384561421 |
| CK_3 | 3904.697480203 | 0.9282834331338 | 10.01409291806 |
| DL_1 | 3243.481538879 | 0.9420425815038 | 9.430631162356 |
| DL_2 | 3554.769413962 | 0.9370791749835 | 9.340880884649 |
| DL_3 | 3567.843271382 | 0.9335528942116 | 9.434950652741 |
| FHF_1 | 3039.255006413 | 0.9437125748501 | 8.427837274803 |
| FHF_2 | 3268.896867125 | 0.9401397205591 | 9.007827292686 |
| FHF_3 | 3908.145420669 | 0.9275981370593 | 9.990056734177 |
| JZ_1 | 3141.410696504 | 0.9396606786427 | 8.749344430441 |
| JZ_2 | 3935.596468743 | 0.9282235528942 | 9.033862144087 |
| JZ_3 | 3124.232257426 | 0.9423087159012 | 9.214503862824 |
| SL_1 | 2926.740393861 | 0.9484431137728 | 9.262813483726 |
| SL_2 | 3275.469899852 | 0.940259481038 | 9.325830746716 |
| SL_3 | 2836.644261642 | 0.9495076513639 | 8.552950827082 |
| ZL_1 | 3007.175269317 | 0.9432069194945 | 8.707885629828 |
| ZL_2 | 4165.741839083 | 0.9273053892216 | 9.803028075383 |
| ZL_3 | 2789.295858023 | 0.9476380572187 | 8.78670511419 |

**Fig 4. Community composition of soil bacteria at the phylum level.**

|  | DL | ZL | SL | FHF | JZ | CK |
| --- | --- | --- | --- | --- | --- | --- |
| p__Tenericutes | 4.43E-05 | 2.22E-05 | 2.22E-05 | 2.22E-05 | 2.22E-05 | 0 |
| p__Candidatus_Berkelbacteria | 4.43E-05 | 0 | 0.000111 | 0.000133 | 6.65E-05 | 2.22E-05 |
| p__Parcubacteria | 0.003414 | 0.001618 | 0.001374 | 0.001818 | 0.004278 | 0.003702 |
| p__Atribacteria | 2.22E-05 | 0 | 0 | 0.000111 | 2.22E-05 | 0 |
| p__Planctomycetes | 0.010086 | 0.012126 | 0.01268 | 0.011062 | 0.010485 | 0.011239 |
| p__Bacteroidetes | 0.031478 | 0.018266 | 0.034781 | 0.024805 | 0.018355 | 0.026468 |
| p__Verrucomicrobia | 0.006318 | 0.006473 | 0.007648 | 0.006273 | 0.012037 | 0.013677 |
| p__Gemmatimonadetes | 0.060229 | 0.081244 | 0.07936 | 0.063976 | 0.053911 | 0.075946 |
| p__Fibrobacteres | 0.000111 | 0.000421 | 0.000111 | 2.22E-05 | 0.000266 | 0.000155 |
| p__Proteobacteria | 0.374609 | 0.296779 | 0.376272 | 0.27552 | 0.275543 | 0.313516 |
| p__WS2 | 0 | 0 | 2.22E-05 | 0.000111 | 0.000133 | 8.87E-05 |
| p__Microgenomates | 8.87E-05 | 2.22E-05 | 4.43E-05 | 0.000333 | 0.000111 | 0.000111 |
| p__Chloroflexi | 0.118086 | 0.22039 | 0.106005 | 0.169604 | 0.236949 | 0.130522 |
| p__SBR1093 | 6.65E-05 | 0.000133 | 0 | 2.22E-05 | 0.000133 | 0.000155 |
| p__Hydrogenedentes | 0 | 0 | 0 | 4.43E-05 | 0 | 0 |
| p__Deferribacteres | 0 | 0.000111 | 0.000732 | 0.000133 | 0.0002 | 0.000244 |
| p__RBG-1_Zixibacteria | 0 | 0 | 0 | 0 | 0 | 2.22E-05 |
| p__Euryarchaeota | 0.000333 | 0.00051 | 0.000466 | 4.43E-05 | 0.002461 | 0.000576 |
| p__Ignavibacteriae | 8.87E-05 | 6.65E-05 | 0 | 0 | 8.87E-05 | 8.87E-05 |
| p__Armatimonadetes | 0.000709 | 0.000399 | 0.000732 | 0.001463 | 0.000621 | 0.001175 |
| p__Acidobacteria | 0.165902 | 0.168252 | 0.097648 | 0.151825 | 0.186806 | 0.202767 |
| p__Omnitrophica | 0 | 0 | 0 | 4.43E-05 | 4.43E-05 | 0.000177 |
| p__Synergistetes | 4.43E-05 | 2.22E-05 | 0.000266 | 0.000887 | 8.87E-05 | 2.22E-05 |
| p__Saccharibacteria | 0.034559 | 0.012347 | 0.012946 | 0.011749 | 0.006362 | 0.007714 |
| p__WS6 | 8.87E-05 | 4.43E-05 | 4.43E-05 | 0.000244 | 2.22E-05 | 0.0002 |
| p__Chlamydiae | 0.001507 | 0.001086 | 0.001153 | 0.000931 | 0.001507 | 0.001485 |
| p__Deinococcus-Thermus | 2.22E-05 | 4.43E-05 | 2.22E-05 | 6.65E-05 | 8.87E-05 | 8.87E-05 |
| p__GAL15 | 0.004411 | 0.00583 | 0.000599 | 0.001042 | 0.005852 | 0.005542 |
| p__Peregrinibacteria | 2.22E-05 | 6.65E-05 | 2.22E-05 | 6.65E-05 | 0.000111 | 8.87E-05 |
| p__Elusimicrobia | 0.000443 | 0.000709 | 0.000576 | 0.001219 | 0.001441 | 0.001818 |
| p__FBP | 0.000111 | 0 | 2.22E-05 | 0 | 2.22E-05 | 0 |
| p__Nitrospirae | 0.005653 | 0.005697 | 0.005675 | 0.004943 | 0.010264 | 0.012746 |
| p__Latescibacteria | 0.000909 | 0.000643 | 0.00051 | 0.00133 | 0.003702 | 0.004589 |
| p__unidentified | 0.005498 | 0.006961 | 0.008025 | 0.00665 | 0.005764 | 0.006074 |
| p__Spirochaetae | 0.000266 | 0.000266 | 0.0002 | 0.000333 | 0.001663 | 0.000732 |
| p__TM6_Dependentiae | 0.00051 | 0.000399 | 0.000488 | 0.000466 | 0.00031 | 0.000399 |
| p__Actinobacteria | 0.13919 | 0.128527 | 0.200084 | 0.14103 | 0.108909 | 0.128926 |
| p__BRC1 | 8.87E-05 | 0 | 6.65E-05 | 0 | 6.65E-05 | 0.000111 |
| p__Lentisphaerae | 4.43E-05 | 0.000111 | 6.65E-05 | 0 | 0 | 0.000111 |
| p__Thaumarchaeota | 0 | 2.22E-05 | 0 | 0 | 4.43E-05 | 0 |
| p__Cyanobacteria | 0.004833 | 0.002039 | 0.008135 | 0.005874 | 0.008978 | 0.011039 |
| p__Gracilibacteria | 0 | 2.22E-05 | 0 | 2.22E-05 | 0 | 0 |
| p__FCPU426 | 0.001574 | 0.000643 | 0.00153 | 0.000443 | 0.001973 | 0.002328 |
| p__Fusobacteria | 0 | 2.22E-05 | 4.43E-05 | 0.00031 | 0 | 0 |
| p__Firmicutes | 0.028286 | 0.027155 | 0.040234 | 0.11454 | 0.039791 | 0.034803 |
| p__Chlorobi | 0.00031 | 0.00051 | 0.001286 | 0.000488 | 0.00051 | 0.000532 |

**Fig 5. Community composition of soil bacteria at the genus level.**

|  | DL | ZL | SL | FHF | JZ | CK |
| --- | --- | --- | --- | --- | --- | --- |
| g__Fastidiosipila | 2.22E-05 | 0 | 2.22E-05 | 0.0002 | 6.65E-05 | 0 |
| g__Pseudomonas | 0.000754 | 0.000443 | 0.002372 | 0.000776 | 0.00399 | 0.001042 |
| g__Dokdonella | 0.000621 | 0.000887 | 0.001352 | 0.000865 | 0.00082 | 0.000798 |
| g__Acetitomaculum | 0.0002 | 0 | 0 | 0 | 0 | 0 |
| g__Jatrophihabitans | 0.002039 | 0.000798 | 0.004966 | 0.001153 | 0.000488 | 0.000931 |
| g__Chryseolinea | 0 | 6.65E-05 | 6.65E-05 | 0.000333 | 0.000222 | 0.000133 |
| g__Ruminofilibacter | 0 | 0 | 2.22E-05 | 0.000133 | 0 | 2.22E-05 |
| g__Megasphaera | 0.000133 | 0 | 0 | 0 | 0 | 0 |
| g__Clostridium_sensu_stricto_7 | 2.22E-05 | 2.22E-05 | 0 | 4.43E-05 | 0 | 4.43E-05 |
| g__G55 | 0 | 2.22E-05 | 6.65E-05 | 4.43E-05 | 2.22E-05 | 2.22E-05 |
| g__Defluviicoccus | 0.000488 | 0.000177 | 0.000133 | 0.0002 | 0.000133 | 6.65E-05 |
| g__Bacillus | 0.00368 | 0.005054 | 0.003525 | 0.007537 | 0.003746 | 0.006894 |
| g__Lachnoclostridium_5 | 4.43E-05 | 0 | 0 | 0 | 0 | 0 |
| g__Fonticella | 2.22E-05 | 0 | 6.65E-05 | 6.65E-05 | 4.43E-05 | 2.22E-05 |
| g__Candidatus_Saccharimonas | 0 | 0 | 2.22E-05 | 0 | 2.22E-05 | 2.22E-05 |
| g__Family_XIII_UCG-002 | 0 | 0 | 0 | 8.87E-05 | 2.22E-05 | 0 |
| g__Stella | 2.22E-05 | 2.22E-05 | 6.65E-05 | 8.87E-05 | 0 | 6.65E-05 |
| g__Kurthia | 0 | 0 | 2.22E-05 | 0 | 6.65E-05 | 0 |
| g__CL500-29_marine_group | 2.22E-05 | 0.000111 | 0.000133 | 0.0002 | 0 | 0.000355 |
| g__Rhizomicrobium | 0.023564 | 0.015717 | 0.022722 | 0.011571 | 0.013367 | 0.015739 |
| g__Weissella | 0 | 0 | 2.22E-05 | 2.22E-05 | 0.003037 | 0 |
| g__metal-contaminated_soil_clone_K20-12 | 0 | 0 | 0 | 4.43E-05 | 0.000155 | 0 |
| g__Salinispora | 0.000133 | 4.43E-05 | 6.65E-05 | 0.000222 | 6.65E-05 | 0.000111 |
| g__Dactylosporangium | 0.00051 | 0.000532 | 0.000377 | 0.000665 | 0.00051 | 0.000355 |
| g__Polaromonas | 2.22E-05 | 2.22E-05 | 0 | 0 | 2.22E-05 | 0 |
| g__Cellvibrio | 0.000399 | 0.000399 | 0.000399 | 0.000333 | 0.000687 | 0.000244 |
| g__Pullulanibacillus | 8.87E-05 | 0.000155 | 8.87E-05 | 0.000466 | 0.000177 | 4.43E-05 |
| g__Christensenellaceae_R-7_group | 0 | 0 | 0 | 0 | 6.65E-05 | 0 |
| g__Ruminococcaceae_UCG-014 | 0.000266 | 0.000266 | 0.000842 | 0.001175 | 0.000266 | 0.000621 |
| g__Clostridium_sensu_stricto_12 | 0.000111 | 4.43E-05 | 2.22E-05 | 2.22E-05 | 6.65E-05 | 0.000111 |
| g__I-8 | 0.000177 | 0 | 0.000177 | 0 | 4.43E-05 | 0 |
| g__Proteus | 2.22E-05 | 2.22E-05 | 8.87E-05 | 2.22E-05 | 8.87E-05 | 2.22E-05 |
| g__Pedobacter | 0.000599 | 0.000488 | 0.001219 | 0.0002 | 0.00051 | 0.000177 |
| g__Nonomuraea | 0.000355 | 0.00031 | 0.000266 | 0.000798 | 0.00031 | 0.000244 |
| g__Cylindrotheca_closterium | 0.000111 | 6.65E-05 | 0.000466 | 0.000399 | 0.000111 | 0.000532 |
| g__Fusobacterium | 0 | 2.22E-05 | 4.43E-05 | 0 | 0 | 0 |
| g__Rikenellaceae_RC9_gut_group | 8.87E-05 | 0.000333 | 0.000798 | 0.000399 | 0.000421 | 0.000466 |
| g__Solirubrobacter | 0.001064 | 0.000399 | 0.00082 | 0.001352 | 0.000909 | 0.000998 |
| g__Aminivibrio | 0 | 2.22E-05 | 2.22E-05 | 0.000288 | 4.43E-05 | 2.22E-05 |
| g__Catenisphaera | 2.22E-05 | 0 | 0 | 0 | 0 | 0 |
| g__Nitrococcus | 0 | 0 | 2.22E-05 | 0 | 0 | 0 |
| g__Nordella | 0.000222 | 0.000111 | 0.000665 | 0.00031 | 0.000333 | 0.000177 |
| g__Gardnerella | 0 | 0 | 0 | 0.000599 | 0 | 0 |
| g__Chitinivorax | 0 | 0 | 0 | 0 | 2.22E-05 | 4.43E-05 |
| g__Candidatus_Microthrix | 8.87E-05 | 8.87E-05 | 0.000111 | 0.0002 | 6.65E-05 | 0.000244 |
| g__agricultural_soil_bacterium_SC-I-84 | 0.000177 | 0.000155 | 0.000222 | 8.87E-05 | 0.000133 | 0.000177 |
| g__Sphingopyxis | 0.000177 | 8.87E-05 | 0.000576 | 0.000244 | 0.000133 | 6.65E-05 |
| g__Faecalibacterium | 0 | 0 | 0 | 2.22E-05 | 0 | 0 |
| g__Acidibacter | 0.009067 | 0.008756 | 0.021613 | 0.008535 | 0.008246 | 0.009709 |
| g__Coprococcus_1 | 0 | 0 | 6.65E-05 | 8.87E-05 | 0.000111 | 4.43E-05 |
| g__Clostridium_sensu_stricto_13 | 2.22E-05 | 0 | 0 | 0 | 0 | 0 |
| g__Aerococcus | 0 | 0 | 0 | 0.000133 | 0 | 0 |
| g__Permianibacter | 0 | 0 | 0 | 0 | 6.65E-05 | 6.65E-05 |
| g__planctomycete_WY108 | 0 | 0 | 0 | 0 | 0 | 2.22E-05 |
| g__Hydrogenophaga | 0.000133 | 2.22E-05 | 4.43E-05 | 0.000111 | 6.65E-05 | 6.65E-05 |
| g__Clostridium_sensu_stricto_1 | 0.000133 | 0.000111 | 0.000377 | 0.003037 | 0.000288 | 0.000488 |
| g__Mizugakiibacter | 0.024938 | 0.005409 | 0.018044 | 0.001862 | 0.001773 | 0.00297 |
| g__Blastocatella | 0 | 0 | 0 | 0 | 4.43E-05 | 0 |
| g__Tyzzerella | 4.43E-05 | 0 | 0 | 2.22E-05 | 0 | 2.22E-05 |
| g__Solimonas | 2.22E-05 | 0 | 0 | 0 | 0 | 0 |
| g__Aeromonas | 6.65E-05 | 2.22E-05 | 0 | 6.65E-05 | 0 | 2.22E-05 |
| g__Peptostreptococcus | 0 | 0 | 0 | 0.000177 | 0 | 2.22E-05 |
| g__Undibacterium | 0 | 2.22E-05 | 0 | 0 | 2.22E-05 | 0 |
| g__Peredibacter | 0 | 0 | 0 | 2.22E-05 | 4.43E-05 | 2.22E-05 |
| g__Acidobacteria_bacterium_WX27 | 0.000177 | 2.22E-05 | 0 | 8.87E-05 | 0 | 0 |
| g__Microvirga | 0.000244 | 0.000155 | 8.87E-05 | 0.000665 | 0.000133 | 0.000355 |
| g__Prevotellaceae_Ga6A1_group | 0 | 0 | 0 | 0 | 2.22E-05 | 0 |
| g__Mucispirillum | 0 | 0.000111 | 0.000732 | 0.000133 | 0.0002 | 0.000244 |
| g__Lachnoclostridium_12 | 2.22E-05 | 2.22E-05 | 2.22E-05 | 0.0002 | 6.65E-05 | 4.43E-05 |
| g__Agromyces | 2.22E-05 | 0.000177 | 0.000111 | 0.000177 | 8.87E-05 | 0.000488 |
| g__Anaerobacterium | 0 | 2.22E-05 | 2.22E-05 | 0 | 2.22E-05 | 2.22E-05 |
| g__Butyricicoccus | 0 | 0 | 6.65E-05 | 2.22E-05 | 0.000111 | 2.22E-05 |
| g__Flavobacterium | 0.000532 | 0.000488 | 0.001352 | 0.000887 | 0.000266 | 0.004921 |
| g__Papillibacter | 0 | 0 | 2.22E-05 | 6.65E-05 | 0 | 0 |
| g__Roseomonas | 4.43E-05 | 2.22E-05 | 6.65E-05 | 0.000111 | 4.43E-05 | 6.65E-05 |
| g__actinobacterium_YJF2-33 | 0 | 2.22E-05 | 0 | 2.22E-05 | 2.22E-05 | 0 |
| g__Thermosporothrix | 0.000532 | 0.001219 | 0.000488 | 0.000488 | 0.000621 | 0.000576 |
| g__Lentibacillus | 0.000532 | 0.000665 | 0.00102 | 0.001042 | 0.0002 | 0.000466 |
| g__Heliimonas | 2.22E-05 | 2.22E-05 | 0 | 2.22E-05 | 2.22E-05 | 6.65E-05 |
| g__candidate_division_WS6_bacterium_GW2011_GWF2_39_15 | 6.65E-05 | 0 | 2.22E-05 | 2.22E-05 | 0 | 0.000177 |
| g__Prevotella_1 | 0 | 0 | 0 | 0 | 2.22E-05 | 0 |
| g__Parabacteroides | 6.65E-05 | 0.000155 | 0.000177 | 0.000288 | 6.65E-05 | 8.87E-05 |
| g__Veillonella | 0 | 0 | 0 | 0 | 0 | 4.43E-05 |
| g__Actinoallomurus | 0.001241 | 6.65E-05 | 6.65E-05 | 0.000111 | 6.65E-05 | 0.00031 |
| g__Actinocatenispora | 0.000266 | 0.000111 | 0.000288 | 0.000177 | 0 | 6.65E-05 |
| g__Intestinimonas | 0 | 2.22E-05 | 8.87E-05 | 2.22E-05 | 4.43E-05 | 2.22E-05 |
| g__Niabella | 4.43E-05 | 2.22E-05 | 0 | 0 | 0 | 0 |
| g__Coxiella | 6.65E-05 | 4.43E-05 | 6.65E-05 | 2.22E-05 | 0.000111 | 4.43E-05 |
| g__Geobacillus | 0.000111 | 2.22E-05 | 6.65E-05 | 0.000222 | 0.000111 | 8.87E-05 |
| g__Thermopolyspora | 0.000798 | 0.000909 | 0.000953 | 0.00133 | 0.000909 | 0.001108 |
| g__Chthoniobacter | 0.000133 | 6.65E-05 | 8.87E-05 | 6.65E-05 | 6.65E-05 | 0.000155 |
| g__Catenulispora | 0.000377 | 0.000355 | 0.001552 | 0.000244 | 0.000377 | 0.000399 |
| g__Taibaiella | 0.001618 | 0.0002 | 0.000288 | 6.65E-05 | 6.65E-05 | 4.43E-05 |
| g__Caproiciproducens | 0 | 0 | 0 | 2.22E-05 | 0 | 0 |
| g__Bauldia | 0.000643 | 0.000621 | 0.000665 | 0.001264 | 0.000687 | 0.000953 |
| g__Ralstonia | 6.65E-05 | 0.000133 | 2.22E-05 | 6.65E-05 | 0.000377 | 0.000621 |
| g__Cronobacter | 0 | 0 | 8.87E-05 | 2.22E-05 | 0.000177 | 0 |
| g__Arachidicoccus | 0.000953 | 4.43E-05 | 4.43E-05 | 0 | 4.43E-05 | 0 |
| g__Peptoniphilus | 0 | 0 | 0 | 8.87E-05 | 0 | 0 |
| g__bacterium_endosymbiont_of_Onthophagus_Taurus | 0 | 0 | 0 | 0 | 0 | 2.22E-05 |
| g__Pseudogulbenkiania | 0.000887 | 0.000333 | 0 | 0.000687 | 2.22E-05 | 0.00051 |
| g__Desulfovirga | 0 | 0 | 0 | 2.22E-05 | 4.43E-05 | 0.000111 |
| g__Leuconostoc | 0 | 0 | 0 | 0 | 0.000576 | 0 |
| g__Brevibacterium | 4.43E-05 | 0 | 2.22E-05 | 0 | 0.000111 | 0 |
| g__Ruminococcaceae_NK4A214_group | 0 | 2.22E-05 | 4.43E-05 | 2.22E-05 | 2.22E-05 | 6.65E-05 |
| g__Psychroglaciecola | 0 | 0 | 0 | 8.87E-05 | 2.22E-05 | 2.22E-05 |
| g__Georgfuchsia | 4.43E-05 | 8.87E-05 | 2.22E-05 | 4.43E-05 | 4.43E-05 | 2.22E-05 |
| g__Candidatus_Yanofskybacteria_bacterium_GW2011_GWA2_41_22 | 0.0002 | 0 | 2.22E-05 | 0 | 6.65E-05 | 2.22E-05 |
| g__Rivibacter | 0 | 0 | 0 | 8.87E-05 | 4.43E-05 | 0.000177 |
| g__Sporichthya | 4.43E-05 | 6.65E-05 | 4.43E-05 | 0 | 0 | 4.43E-05 |
| g__Bromus_tectorum | 2.22E-05 | 0 | 0 | 0 | 2.22E-05 | 0 |
| g__Camelimonas | 0 | 4.43E-05 | 0 | 2.22E-05 | 0 | 2.22E-05 |
| g__Solobacterium | 8.87E-05 | 0 | 0 | 2.22E-05 | 0 | 0 |
| g__Andreprevotia | 0 | 0.000111 | 0.0002 | 2.22E-05 | 0 | 2.22E-05 |
| g__Pseudogracilibacillus | 2.22E-05 | 6.65E-05 | 4.43E-05 | 6.65E-05 | 4.43E-05 | 0 |
| g__Vicinamibacter | 2.22E-05 | 2.22E-05 | 0 | 0 | 0.000133 | 4.43E-05 |
| g__Aridibacter | 0 | 0 | 2.22E-05 | 0 | 2.22E-05 | 4.43E-05 |
| g__Fluviicola | 0.000687 | 6.65E-05 | 0.000333 | 8.87E-05 | 8.87E-05 | 0 |
| g__Prevotellaceae_UCG-001 | 2.22E-05 | 0.000133 | 0.000732 | 0.000244 | 0.000177 | 0.000576 |
| g__Cellulosimicrobium | 0.000266 | 0.000266 | 0.000377 | 0.000266 | 6.65E-05 | 0.000576 |
| g__Acinetobacter | 6.65E-05 | 0 | 0 | 2.22E-05 | 6.65E-05 | 0 |
| g__Arcticibacter | 0.0002 | 2.22E-05 | 2.22E-05 | 0 | 0 | 0 |
| g__Bacillaria_paxillifer | 0.000111 | 4.43E-05 | 2.22E-05 | 8.87E-05 | 8.87E-05 | 6.65E-05 |
| g__Candidatus_Koribacter | 0.001397 | 0.002283 | 0.001397 | 0.002882 | 0.001219 | 0.005409 |
| g__Neisseria | 0 | 4.43E-05 | 4.43E-05 | 0 | 6.65E-05 | 0 |
| g__Geminicoccus | 6.65E-05 | 2.22E-05 | 0 | 8.87E-05 | 2.22E-05 | 2.22E-05 |
| g__Tahibacter | 6.65E-05 | 0.000155 | 2.22E-05 | 0.000222 | 8.87E-05 | 0.000177 |
| g__Duganella | 0.000133 | 0.000399 | 0.001175 | 0.000865 | 0.000532 | 0.000842 |
| g__Tuberibacillus | 0.000643 | 0.000421 | 0.000466 | 0.001485 | 0.000288 | 0.00031 |
| g__Herpetosiphon | 2.22E-05 | 0 | 0 | 2.22E-05 | 0 | 0.000111 |
| g__unidentified | 0.592317 | 0.675046 | 0.578329 | 0.643945 | 0.683847 | 0.643413 |
| g__Blastochloris | 2.22E-05 | 0 | 4.43E-05 | 0 | 0 | 4.43E-05 |
| g__Massilia | 0.000975 | 0.00133 | 0.002305 | 0.003879 | 0.001574 | 0.001241 |
| g__Castellaniella | 0.000532 | 0 | 0 | 2.22E-05 | 0 | 0 |
| g__PAUC26f | 0.001241 | 0.001995 | 8.87E-05 | 0.000288 | 0.002128 | 0.001374 |
| g__Pseudoclavibacter | 0 | 0 | 0 | 0 | 6.65E-05 | 0 |
| g__Pandoraea | 4.43E-05 | 0 | 0 | 0.000111 | 0 | 0 |
| g__Saccharofermentans | 0 | 0 | 2.22E-05 | 2.22E-05 | 8.87E-05 | 0 |
| g__Azospirillum | 4.43E-05 | 0 | 0 | 0.000222 | 0 | 6.65E-05 |
| g__Lachnospiraceae_UCG-006 | 0.00051 | 0.000754 | 0.000953 | 0.000576 | 0.000333 | 0.000466 |
| g__Polycyclovorans | 0 | 4.43E-05 | 6.65E-05 | 0 | 0.000133 | 8.87E-05 |
| g__Ornithinicoccus | 0 | 0 | 2.22E-05 | 0 | 0 | 2.22E-05 |
| g__Phycisphaera | 0 | 4.43E-05 | 8.87E-05 | 0.000133 | 0.000554 | 0.000111 |
| g__Rudaea | 0 | 2.22E-05 | 0 | 2.22E-05 | 0 | 0 |
| g__Polyangium | 2.22E-05 | 0.000111 | 0.0002 | 0.000133 | 2.22E-05 | 0.000177 |
| g__Gemmatirosa | 0.001507 | 0.00031 | 0.000554 | 0.000887 | 0.001175 | 0.00102 |
| g__Fimbriimonas | 2.22E-05 | 0 | 2.22E-05 | 0 | 2.22E-05 | 0 |
| g__Marvinbryantia | 4.43E-05 | 2.22E-05 | 8.87E-05 | 0.0002 | 6.65E-05 | 0.000155 |
| g__Streptosporangium | 2.22E-05 | 0 | 2.22E-05 | 0 | 0 | 2.22E-05 |
| g__Amaricoccus | 0 | 2.22E-05 | 0 | 2.22E-05 | 0 | 6.65E-05 |
| g__Pediococcus | 0 | 2.22E-05 | 0 | 6.65E-05 | 2.22E-05 | 0 |
| g__Woodsholea | 0.000355 | 0.000222 | 0.000399 | 0.000687 | 0.000443 | 0.000687 |
| g__Pedosphaera | 4.43E-05 | 2.22E-05 | 0 | 2.22E-05 | 0 | 0 |
| g__Sulfurovum | 2.22E-05 | 0 | 2.22E-05 | 0.000222 | 0 | 2.22E-05 |
| g__Acidocella | 0 | 8.87E-05 | 8.87E-05 | 0.000222 | 8.87E-05 | 0.000111 |
| g__Desulfitibacter | 0 | 0 | 4.43E-05 | 0 | 0 | 0 |
| g__Gaiella | 0.00153 | 0.001485 | 0.000798 | 0.001685 | 0.001552 | 0.002062 |
| g__Saccharothrix | 2.22E-05 | 0.000111 | 6.65E-05 | 8.87E-05 | 0.00031 | 0.000177 |
| g__Dongia | 0.002372 | 0.002217 | 0.003724 | 0.002438 | 0.002438 | 0.002372 |
| g__Eubacterium_ruminantium_group | 0.000133 | 0.000222 | 0.000222 | 0.000377 | 0 | 0.0002 |
| g__Serratia | 0 | 2.22E-05 | 0 | 0 | 6.65E-05 | 0 |
| g__Alistipes | 0.000222 | 0.000244 | 0.00184 | 0.000732 | 0.000576 | 0.000931 |
| g__Oceanobacillus | 0.000333 | 0.000399 | 0.000532 | 0.000931 | 0.000177 | 0.000333 |
| g__Ruminococcaceae_UCG-005 | 0 | 0 | 0.000155 | 0 | 0 | 0 |
| g__Comamonas | 4.43E-05 | 2.22E-05 | 0.001906 | 8.87E-05 | 6.65E-05 | 6.65E-05 |
| g__Parafilimonas | 0.000111 | 0.000155 | 8.87E-05 | 0.000133 | 4.43E-05 | 6.65E-05 |
| g__Thermomonospora | 8.87E-05 | 0.0002 | 0.000111 | 0.00031 | 0.000133 | 0.0002 |
| g__Pseudonocardia | 0.001153 | 0.000621 | 0.000643 | 0.00133 | 0.000687 | 0.000931 |
| g__Pedomicrobium | 0.000399 | 0.000377 | 0.000333 | 0.000576 | 0.000643 | 0.000754 |
| g__Methylophilus | 6.65E-05 | 8.87E-05 | 8.87E-05 | 2.22E-05 | 0 | 4.43E-05 |
| g__Vulgatibacter | 0.000111 | 0.000155 | 0.000111 | 0.000266 | 0.000133 | 0.000244 |
| g__Minicystis | 4.43E-05 | 2.22E-05 | 2.22E-05 | 4.43E-05 | 4.43E-05 | 0.000244 |
| g__Anaerotruncus | 0 | 8.87E-05 | 0.000266 | 0.000155 | 0.000155 | 0.000111 |
| g__Faecalibaculum | 2.22E-05 | 2.22E-05 | 6.65E-05 | 8.87E-05 | 6.65E-05 | 2.22E-05 |
| g__Acidicaldus | 0.000953 | 0.000355 | 0.000909 | 0.000288 | 0.000111 | 0.000466 |
| g__Chitinophaga | 0.000377 | 0.00102 | 0.000554 | 0.000222 | 2.22E-05 | 0.000177 |
| g__Persicaria_minor | 4.43E-05 | 2.22E-05 | 2.22E-05 | 2.22E-05 | 2.22E-05 | 0 |
| g__candidate_division_TM7_bacterium_LY2 | 6.65E-05 | 4.43E-05 | 0 | 4.43E-05 | 4.43E-05 | 2.22E-05 |
| g__Sphaerisporangium | 4.43E-05 | 0 | 0 | 4.43E-05 | 0 | 6.65E-05 |
| g__Aquicella | 0.001219 | 0.001419 | 0.001685 | 0.00235 | 0.002882 | 0.00215 |
| g__Nubsella | 8.87E-05 | 0 | 0.001751 | 4.43E-05 | 2.22E-05 | 0 |
| g__Rhodococcus | 2.22E-05 | 0 | 0 | 0 | 0 | 0 |
| g__Singulisphaera | 0.001884 | 0.00215 | 0.00133 | 0.001552 | 0.001596 | 0.001241 |
| g__Flavitalea | 0.000554 | 0.0002 | 0.000466 | 0.000377 | 0.000266 | 0.000222 |
| g__Geodermatophilus | 0.002062 | 0.000599 | 0.001929 | 0.001397 | 0.00031 | 0.000776 |
| g__Wandonia | 0.00051 | 0 | 0 | 0 | 0 | 0 |
| g__Acidothermus | 0.031833 | 0.028596 | 0.048702 | 0.016714 | 0.035623 | 0.026003 |
| g__Crenotalea | 0.00215 | 2.22E-05 | 0.001197 | 2.22E-05 | 6.65E-05 | 0 |
| g__Luteibacter | 0.026091 | 0.000887 | 0.000488 | 0.000931 | 0.001241 | 0.0002 |
| g__Ruminiclostridium | 2.22E-05 | 0.000111 | 0.000488 | 6.65E-05 | 6.65E-05 | 0.000111 |
| g__Clostridium_sensu_stricto_18 | 0 | 0 | 0 | 0 | 0 | 2.22E-05 |
| g__Ruminococcus_gauvreauii_group | 0.0002 | 0 | 0 | 0 | 0 | 0 |
| g__Candidatus_Methylacidiphilum | 0 | 0 | 0 | 0 | 2.22E-05 | 0 |
| g__Blautia | 0 | 4.43E-05 | 0 | 0 | 8.87E-05 | 2.22E-05 |
| g__Fibrobacter | 0 | 0 | 0 | 0 | 2.22E-05 | 0 |
| g__Longispora | 2.22E-05 | 0 | 0 | 0 | 0 | 2.22E-05 |
| g__Desulfovibrio | 2.22E-05 | 0 | 4.43E-05 | 4.43E-05 | 2.22E-05 | 0 |
| g__Chitinimonas | 4.43E-05 | 0.000155 | 0.000155 | 8.87E-05 | 0.000177 | 0.000244 |
| g__Caldibacillus | 0.000111 | 2.22E-05 | 4.43E-05 | 0.000288 | 0 | 8.87E-05 |
| g__Delftia | 0 | 0 | 0.001618 | 8.87E-05 | 6.65E-05 | 2.22E-05 |
| g__Adlercreutzia | 2.22E-05 | 0 | 0 | 2.22E-05 | 4.43E-05 | 0 |
| g__Candidatus_Captivus | 0 | 0.000111 | 4.43E-05 | 6.65E-05 | 4.43E-05 | 0 |
| g__Actinomadura | 0.000599 | 0.000554 | 0.00082 | 0.001153 | 0.000621 | 0.001241 |
| g__Family_XIII_UCG-001 | 0 | 0 | 2.22E-05 | 0 | 2.22E-05 | 0 |
| g__Rhizobium | 0.000333 | 0.000842 | 0.000244 | 0.000599 | 0.0002 | 0.000754 |
| g__Prevotella_7 | 0 | 0 | 4.43E-05 | 0 | 2.22E-05 | 0 |
| g__Bilophila | 6.65E-05 | 0 | 6.65E-05 | 0.000111 | 4.43E-05 | 0 |
| g__Williamsia | 0 | 0 | 0 | 0 | 2.22E-05 | 2.22E-05 |
| g__Vogesella | 4.43E-05 | 4.43E-05 | 0 | 4.43E-05 | 0 | 0.000111 |
| g__Pseudobacteroides | 0 | 0 | 2.22E-05 | 0 | 0 | 0 |
| g__Anaeromyxobacter | 0.000709 | 0.000975 | 0.000443 | 0.001352 | 0.000754 | 0.002106 |
| g__Terrimicrobium | 0 | 2.22E-05 | 0 | 4.43E-05 | 0 | 2.22E-05 |
| g__Holophaga | 0.000333 | 0.001441 | 0.000443 | 0.001441 | 0.000643 | 0.001153 |
| g__Amycolatopsis | 0.001153 | 0.002372 | 0.003325 | 0.000998 | 0.000266 | 0.000466 |
| g__Clostridium_sensu_stricto_3 | 0.000133 | 8.87E-05 | 0 | 6.65E-05 | 2.22E-05 | 8.87E-05 |
| g__Phenylobacterium | 0.002793 | 0.002195 | 0.002416 | 0.003015 | 0.001773 | 0.002882 |
| g__Peptococcus | 0 | 0 | 0 | 2.22E-05 | 2.22E-05 | 2.22E-05 |
| g__Desulfosporosinus | 2.22E-05 | 0 | 0 | 2.22E-05 | 0.000133 | 2.22E-05 |
| g__Tetraphis_pellucida | 0 | 0 | 2.22E-05 | 4.43E-05 | 0 | 0 |
| g__Skermanella | 2.22E-05 | 2.22E-05 | 0 | 2.22E-05 | 4.43E-05 | 2.22E-05 |
| g__Nitrospira | 0.004256 | 0.003148 | 0.005165 | 0.003901 | 0.007759 | 0.008978 |
| g__Planifilum | 0 | 2.22E-05 | 2.22E-05 | 0 | 2.22E-05 | 0 |
| g__Sphingomonas | 0.014342 | 0.011594 | 0.012658 | 0.020882 | 0.0092 | 0.010574 |
| g__Hyphomicrobium | 0.001241 | 0.001042 | 0.000909 | 0.001153 | 0.000754 | 0.000887 |
| g__Ruminococcaceae_UCG-009 | 0 | 2.22E-05 | 8.87E-05 | 0 | 2.22E-05 | 0 |
| g__Longimycelium | 0.000399 | 0.000244 | 0.001507 | 0.0002 | 6.65E-05 | 0.000177 |
| g__DS-100 | 2.22E-05 | 4.43E-05 | 0 | 4.43E-05 | 0.000111 | 0.0002 |
| g__Nakamurella | 0.000776 | 0.0002 | 0.000266 | 0.000377 | 6.65E-05 | 0.000443 |
| g__Ramlibacter | 0.000621 | 0.000665 | 0.000266 | 0.001219 | 0.000355 | 0.000998 |
| g__Nitrosospira | 0.000776 | 6.65E-05 | 0.000177 | 0.000111 | 0.000244 | 8.87E-05 |
| g__Kroppenstedtia | 0.00051 | 0.00082 | 0.000842 | 0.001131 | 0.00051 | 0.00051 |
| g__Dialister | 0 | 0 | 0 | 0.0002 | 0 | 0 |
| g__Devosia | 0.004411 | 0.003236 | 0.003325 | 0.003635 | 0.002017 | 0.003059 |
| g__Candidatus_Odyssella | 0 | 2.22E-05 | 0.000133 | 0.000333 | 4.43E-05 | 6.65E-05 |
| g__Pusillimonas | 0.00051 | 0 | 4.43E-05 | 0.000133 | 4.43E-05 | 4.43E-05 |
| g__Sporolactobacillus | 0.000133 | 0 | 0 | 0 | 0 | 0 |
| g__Terribacillus | 4.43E-05 | 0 | 0 | 0 | 0 | 2.22E-05 |
| g__Haliangium | 0.005453 | 0.009709 | 0.013012 | 0.010574 | 0.006916 | 0.018399 |
| g__Azohydromonas | 8.87E-05 | 0.000177 | 0.000155 | 0.000488 | 0.000177 | 0.000421 |
| g__Bacteroides | 2.22E-05 | 0.000443 | 0.002039 | 0.000998 | 0.000576 | 0.000709 |
| g__Hydrogenispora | 8.87E-05 | 0.0002 | 8.87E-05 | 0.000177 | 0.000177 | 0.000133 |
| g__Lachnoclostridium | 0.0002 | 0.000288 | 0.000665 | 0.000554 | 0.00031 | 0.000355 |
| g__Nannocystis | 4.43E-05 | 0 | 2.22E-05 | 0 | 0 | 0 |
| g__Aneurinibacillus | 2.22E-05 | 2.22E-05 | 0 | 6.65E-05 | 2.22E-05 | 0 |
| g__Acidobacterium | 0.006872 | 0.002217 | 0.008446 | 0.002993 | 0.000953 | 0.003081 |
| g__Clostridium_sensu_stricto_10 | 6.65E-05 | 0.000111 | 2.22E-05 | 2.22E-05 | 8.87E-05 | 0.0002 |
| g__Lachnospiraceae_UCG-001 | 0 | 0.000133 | 0.000421 | 0.000133 | 0.0002 | 0.000155 |
| g__Lautropia | 2.22E-05 | 0 | 0 | 4.43E-05 | 2.22E-05 | 0 |
| g__Burkholderia-Paraburkholderia | 0.004301 | 0.007936 | 0.003502 | 0.002571 | 0.001241 | 0.002793 |
| g__Anaerobacillus | 2.22E-05 | 0.0002 | 0.000155 | 0.000599 | 0.0002 | 0.0002 |
| g__Pectobacterium | 8.87E-05 | 0.000288 | 0 | 0 | 2.22E-05 | 0 |
| g__Caldicoprobacter | 6.65E-05 | 2.22E-05 | 0.0002 | 0.000532 | 0 | 0.000111 |
| g__Fodinicola | 6.65E-05 | 0.000133 | 6.65E-05 | 0.000687 | 0.000133 | 0.00031 |
| g__Ktedonobacter | 0.000377 | 0.000443 | 0.0002 | 0.00051 | 0.000532 | 0.000288 |
| g__Crossiella | 0.000266 | 0.000133 | 0.001663 | 8.87E-05 | 0.000155 | 4.43E-05 |
| g__Achromobacter | 8.87E-05 | 0.000244 | 0 | 2.22E-05 | 0.000377 | 8.87E-05 |
| g__Stenotrophomonas | 8.87E-05 | 0 | 0.002682 | 2.22E-05 | 0.000155 | 4.43E-05 |
| g__OM27_clade | 0.000399 | 0.000488 | 0.000288 | 0.000665 | 0.00031 | 0.000532 |
| g__Haloactinopolyspora | 0.000111 | 2.22E-05 | 6.65E-05 | 0.000111 | 2.22E-05 | 0.000177 |
| g__bacterium_Ellin6537 | 8.87E-05 | 0.000222 | 0.000155 | 0.00031 | 0.000177 | 0.000333 |
| g__Mesorhizobium | 0.002638 | 0.001286 | 0.001219 | 0.00164 | 0.000599 | 0.001042 |
| g__Rhodobacter | 0 | 0 | 0 | 8.87E-05 | 6.65E-05 | 8.87E-05 |
| g__Tepidimicrobium | 6.65E-05 | 0 | 6.65E-05 | 2.22E-05 | 2.22E-05 | 0 |
| g__11-24 | 2.22E-05 | 4.43E-05 | 0 | 0.000111 | 0.000155 | 0.000244 |
| g__Geobacter | 4.43E-05 | 6.65E-05 | 0 | 4.43E-05 | 0.0002 | 0.000709 |
| g__marine_metagenome | 6.65E-05 | 2.22E-05 | 0 | 4.43E-05 | 2.22E-05 | 0 |
| g__Myroides | 0 | 0 | 0 | 0 | 8.87E-05 | 0 |
| g__Roseiflexus | 0.001219 | 0.000887 | 0.000887 | 0.00317 | 0.001374 | 0.002438 |
| g__Butyricimonas | 2.22E-05 | 0 | 0.000355 | 6.65E-05 | 0.000133 | 8.87E-05 |
| g__Paracoccus | 2.22E-05 | 0 | 0 | 2.22E-05 | 0 | 0 |
| g__Parvimonas | 0 | 0 | 0 | 2.22E-05 | 2.22E-05 | 2.22E-05 |
| g__Inquilinus | 0.000576 | 0.000443 | 0.000377 | 0.000266 | 0.000222 | 0.000244 |
| g__Thiobacillus | 2.22E-05 | 0.000155 | 0 | 4.43E-05 | 0 | 0 |
| g__Cellulosibacter | 2.22E-05 | 0 | 2.22E-05 | 0 | 0 | 0 |
| g__Eubacterium_xylanophilum_group | 2.22E-05 | 8.87E-05 | 0.000155 | 4.43E-05 | 0.00031 | 0.000111 |
| g__Azospira | 2.22E-05 | 0 | 0 | 0 | 2.22E-05 | 0 |
| g__Parvibaculum | 0.000133 | 4.43E-05 | 0.000177 | 0.000111 | 0.000111 | 0.000133 |
| g__Alloprevotella | 0 | 0 | 0.000621 | 0.000155 | 4.43E-05 | 0.000111 |
| g__Granulicella | 0.000798 | 0.000754 | 0.001131 | 0.001108 | 0.000443 | 0.000887 |
| g__Rhodanobacter | 0.014675 | 0.007337 | 0.017535 | 0.006983 | 0.0045 | 0.006185 |
| g__Rhodoblastus | 2.22E-05 | 0 | 0 | 0.000155 | 2.22E-05 | 2.22E-05 |
| g__Staphylococcus | 2.22E-05 | 2.22E-05 | 8.87E-05 | 6.65E-05 | 0.000222 | 0.0002 |
| g__Sandaracinus | 8.87E-05 | 0.000421 | 2.22E-05 | 0.000222 | 0.000244 | 0.00051 |
| g__Brevundimonas | 0.000177 | 8.87E-05 | 0.000266 | 0.000111 | 0.000133 | 4.43E-05 |
| g__Actinomyces | 0 | 0 | 2.22E-05 | 0 | 0 | 2.22E-05 |
| g__Variovorax | 0.000532 | 0.000355 | 8.87E-05 | 0.000399 | 0.00031 | 0.000488 |
| g__Luteimonas | 0.000887 | 0.00082 | 0.000266 | 0.000865 | 0.000709 | 0.000443 |
| g__Candidimonas | 2.22E-05 | 0 | 0 | 2.22E-05 | 2.22E-05 | 0 |
| g__Constrictibacter | 0 | 0 | 0 | 2.22E-05 | 0 | 0 |
| g__Luteolibacter | 2.22E-05 | 6.65E-05 | 0.000377 | 6.65E-05 | 8.87E-05 | 4.43E-05 |
| g__Seinonella | 4.43E-05 | 0 | 0 | 0 | 0 | 0 |
| g__Saccharopolyspora | 8.87E-05 | 4.43E-05 | 2.22E-05 | 0.000111 | 8.87E-05 | 6.65E-05 |
| g__Rhodovastum | 0.000887 | 2.22E-05 | 2.22E-05 | 2.22E-05 | 6.65E-05 | 0 |
| g__Acidisoma | 0.000177 | 4.43E-05 | 8.87E-05 | 0.000177 | 6.65E-05 | 0.000532 |
| g__BD1-7_clade | 0 | 4.43E-05 | 2.22E-05 | 0 | 2.22E-05 | 4.43E-05 |
| g__Ilumatobacter | 6.65E-05 | 0.000466 | 0.000133 | 0.000355 | 0.000111 | 0.000266 |
| g__Shinella | 0.000244 | 0.000155 | 0.000133 | 4.43E-05 | 4.43E-05 | 4.43E-05 |
| g__Alcanivorax | 0.002283 | 0 | 0 | 0 | 2.22E-05 | 0 |
| g__Olsenella | 0.00102 | 0 | 0 | 0 | 0 | 0 |
| g__Lachnospiraceae_FCS020_group | 2.22E-05 | 8.87E-05 | 4.43E-05 | 6.65E-05 | 6.65E-05 | 8.87E-05 |
| g__Candidatus_Xiphinematobacter | 4.43E-05 | 0 | 0 | 0 | 0.000133 | 0 |
| g__Chloromonas_perforata | 4.43E-05 | 0 | 2.22E-05 | 2.22E-05 | 4.43E-05 | 2.22E-05 |
| g__Reyranella | 0.001929 | 0.001219 | 0.002549 | 0.001574 | 0.00133 | 0.002394 |
| g__Alterococcus | 0 | 0 | 0 | 0 | 2.22E-05 | 0 |
| g__Pseudohongiella | 2.22E-05 | 8.87E-05 | 0 | 0.000133 | 6.65E-05 | 6.65E-05 |
| g__Mucilaginibacter | 0.001153 | 0.001286 | 0.000621 | 0.000466 | 0.000355 | 0.000443 |
| g__Neochlamydia | 0.000244 | 0.000111 | 0.0002 | 4.43E-05 | 4.43E-05 | 0.000155 |
| g__bacterium_Ellin6543 | 0.00102 | 0.002195 | 0.000355 | 0.001574 | 0.001153 | 0.000887 |
| g__Parviterribacter | 4.43E-05 | 2.22E-05 | 4.43E-05 | 0.000133 | 2.22E-05 | 4.43E-05 |
| g__Gordonia | 4.43E-05 | 2.22E-05 | 0.000111 | 4.43E-05 | 0 | 0 |
| g__Methyloversatilis | 2.22E-05 | 0 | 2.22E-05 | 0 | 0 | 0 |
| g__Spirochaeta_2 | 0.000222 | 0.000222 | 0.0002 | 0.000155 | 0.001596 | 0.000576 |
| g__Microlunatus | 2.22E-05 | 2.22E-05 | 0 | 0 | 0 | 2.22E-05 |
| g__Candidatus_Finniella | 0 | 0 | 2.22E-05 | 0 | 0 | 0 |
| g__Oligoflexus | 0 | 0 | 6.65E-05 | 0.000177 | 0.000177 | 6.65E-05 |
| g__Aciditerrimonas | 0.001929 | 0.002106 | 0.002394 | 0.001507 | 0.001264 | 0.001507 |
| g__Nesterenkonia | 0 | 0 | 0 | 0 | 4.43E-05 | 0 |
| g__Patulibacter | 0.000244 | 0.000244 | 0.0002 | 0.000643 | 0.000244 | 0.000554 |
| g__Pseudolabrys | 0.00716 | 0.004677 | 0.006318 | 0.005187 | 0.003059 | 0.005276 |
| g__Syntrophobacter | 2.22E-05 | 0 | 2.22E-05 | 0 | 0 | 4.43E-05 |
| g__Truepera | 2.22E-05 | 4.43E-05 | 2.22E-05 | 6.65E-05 | 8.87E-05 | 8.87E-05 |
| g__Alkanindiges | 0.000155 | 8.87E-05 | 0.006052 | 4.43E-05 | 2.22E-05 | 2.22E-05 |
| g__Ruminiclostridium_5 | 0 | 4.43E-05 | 0.000133 | 0.000155 | 4.43E-05 | 6.65E-05 |
| g__Leptolyngbya | 0 | 0 | 0 | 0.000111 | 4.43E-05 | 8.87E-05 |
| g__Propionibacterium | 0 | 0 | 0 | 2.22E-05 | 2.22E-05 | 0 |
| g__Sorangium | 0.002261 | 0.005874 | 0.004256 | 0.00348 | 0.003879 | 0.007759 |
| g__Succinivibrio | 0 | 0 | 0 | 2.22E-05 | 2.22E-05 | 0 |
| g__Chthonomonas | 8.87E-05 | 4.43E-05 | 2.22E-05 | 0.000133 | 0.000133 | 0.000133 |
| g__Klebsiella | 0.000399 | 4.43E-05 | 0.000488 | 2.22E-05 | 0.00051 | 0.000222 |
| g__Sediminibacterium | 0.000111 | 0.000222 | 6.65E-05 | 0.000244 | 0.000266 | 0.000177 |
| g__Caulobacter | 0.000244 | 8.87E-05 | 4.43E-05 | 6.65E-05 | 0.000177 | 2.22E-05 |
| g__Subdoligranulum | 0 | 0 | 0 | 2.22E-05 | 0 | 0 |
| g__Dinghuibacter | 0 | 4.43E-05 | 0 | 0 | 0 | 0 |
| g__Blastococcus | 0.000754 | 0.000222 | 8.87E-05 | 0.000244 | 0.000466 | 0.00031 |
| g__Edaphobacter | 0.00031 | 0.000111 | 8.87E-05 | 0.000155 | 2.22E-05 | 0.000111 |
| g__Glycomyces | 2.22E-05 | 0.0002 | 2.22E-05 | 0 | 0 | 2.22E-05 |
| g__Proteiniclasticum | 0.000133 | 0 | 2.22E-05 | 0.00031 | 4.43E-05 | 0 |
| g__Leifsonia | 0.000554 | 0.000754 | 0.000665 | 0.000488 | 0.000266 | 0.000687 |
| g__Humibacter | 0.000865 | 0.000111 | 0.000443 | 0.000133 | 0.000111 | 6.65E-05 |
| g__Brevibacillus | 0.000133 | 0.000111 | 6.65E-05 | 0.000111 | 0.000111 | 0.000288 |
| g__Smaragdicoccus | 0 | 8.87E-05 | 0 | 0 | 0 | 2.22E-05 |
| g__Chlamydomonas_moewusii | 0 | 0 | 6.65E-05 | 0 | 0 | 0 |
| g__Paenibacillus | 0.001086 | 0.000576 | 0.000532 | 0.001596 | 0.000643 | 0.000754 |
| g__groundwater_metagenome | 0.000754 | 0.000155 | 0.000288 | 8.87E-05 | 0.000266 | 8.87E-05 |
| g__Petrimonas | 4.43E-05 | 2.22E-05 | 0.000133 | 0.00133 | 6.65E-05 | 2.22E-05 |
| g__Azospirillum_sp._enrichment_culture_clone_yan50 | 0 | 0 | 0 | 2.22E-05 | 0 | 0 |
| g__Prevotella_6 | 0 | 0 | 0 | 6.65E-05 | 0 | 0 |
| g__Mumia | 2.22E-05 | 0 | 0 | 0 | 0 | 0 |
| g__Ureibacillus | 4.43E-05 | 2.22E-05 | 0.000111 | 0.000133 | 6.65E-05 | 4.43E-05 |
| g__Thermobifida | 2.22E-05 | 2.22E-05 | 0 | 0 | 0 | 2.22E-05 |
| g__Olivibacter | 2.22E-05 | 2.22E-05 | 0 | 0 | 0 | 0 |
| g__Haloferula | 0.000111 | 6.65E-05 | 0.000288 | 4.43E-05 | 4.43E-05 | 2.22E-05 |
| g__Mobilitalea | 0 | 2.22E-05 | 2.22E-05 | 8.87E-05 | 0 | 0 |
| g__Ezakiella | 0 | 0 | 0 | 4.43E-05 | 0 | 0 |
| g__Clostridium_sensu_stricto_5 | 2.22E-05 | 2.22E-05 | 0 | 2.22E-05 | 0 | 0 |
| g__Escherichia-Shigella | 0.000222 | 0.000687 | 0.000288 | 0.000222 | 0.00031 | 0.000155 |
| g__Pirellula | 0 | 0 | 0 | 0 | 0 | 2.22E-05 |
| g__Hamadaea | 0.0002 | 0.000333 | 0.000732 | 0.000222 | 0.00031 | 0.000222 |
| g__Novosphingobium | 0.000177 | 6.65E-05 | 8.87E-05 | 0.000133 | 0.000111 | 6.65E-05 |
| g__Flavisolibacter | 0.000754 | 0.000177 | 0.000288 | 0.000732 | 0.000377 | 0.000687 |
| g__Gemmatimonas | 0.014852 | 0.010929 | 0.014342 | 0.020616 | 0.010995 | 0.018842 |
| g__Phaselicystis | 4.43E-05 | 0.000222 | 2.22E-05 | 0.000443 | 0.000155 | 0.000776 |
| g__Polycladomyces | 0 | 0 | 0 | 0 | 0 | 2.22E-05 |
| g__Corynebacterium_1 | 0 | 0 | 0 | 4.43E-05 | 0.000244 | 8.87E-05 |
| g__Enterococcus | 4.43E-05 | 0 | 0.000155 | 4.43E-05 | 0.000399 | 8.87E-05 |
| g__Sulfobacillus | 0.000111 | 0 | 2.22E-05 | 0 | 0 | 0 |
| g__Thermovum | 0 | 0 | 2.22E-05 | 0 | 0 | 0 |
| g__Cupriavidus | 6.65E-05 | 0.00031 | 0.000111 | 0.000244 | 0.000155 | 0.000488 |
| g__Dyella | 0.01248 | 0.005453 | 0.007958 | 0.006695 | 0.003414 | 0.001818 |
| g__Leptospira | 0 | 0 | 0 | 2.22E-05 | 0 | 2.22E-05 |
| g__Brachybacterium | 0 | 0 | 0 | 0 | 0.000111 | 0 |
| g__Symbiobacterium | 0.000488 | 0.000421 | 0.000709 | 0.000754 | 0.000266 | 0.000532 |
| g__Shimazuella | 4.43E-05 | 0 | 6.65E-05 | 0 | 2.22E-05 | 8.87E-05 |
| g__Prosthecobacter | 8.87E-05 | 2.22E-05 | 0.000177 | 6.65E-05 | 8.87E-05 | 0 |
| g__Eubacterium_nodatum_group | 0.000177 | 4.43E-05 | 0 | 2.22E-05 | 2.22E-05 | 2.22E-05 |
| g__Kaistia | 2.22E-05 | 0 | 0 | 0 | 4.43E-05 | 6.65E-05 |
| g__Ohtaekwangia | 6.65E-05 | 8.87E-05 | 0 | 0.000133 | 0.000222 | 8.87E-05 |
| g__Exiguobacterium | 0 | 6.65E-05 | 0 | 2.22E-05 | 0 | 0 |
| g__Ruminiclostridium_6 | 4.43E-05 | 6.65E-05 | 0.000599 | 0.000244 | 2.22E-05 | 8.87E-05 |
| g__Agaricicola | 0.001064 | 0.000244 | 0.000842 | 0.000155 | 0.000133 | 8.87E-05 |
| g__Turicibacter | 0.0002 | 0.000266 | 0.000687 | 0.001153 | 0 | 0.000288 |
| g__Ammoniphilus | 0.000377 | 0.000155 | 0.0002 | 0.000333 | 0.000576 | 0.000266 |
| g__Rhodopila | 0.000111 | 0.000133 | 6.65E-05 | 8.87E-05 | 8.87E-05 | 0.0002 |
| g__Oryzihumus | 0.000111 | 8.87E-05 | 0 | 0.000155 | 6.65E-05 | 2.22E-05 |
| g__Bifidobacterium | 0.000621 | 0.000155 | 0.000532 | 0.000133 | 0.000732 | 8.87E-05 |
| g__Longilinea | 0 | 0 | 0 | 4.43E-05 | 0 | 0 |
| g__Streptomyces | 0.004943 | 0.008401 | 0.002749 | 0.005209 | 0.002461 | 0.004034 |
| g__Ruminococcaceae_UCG-010 | 0 | 2.22E-05 | 0 | 2.22E-05 | 0 | 0 |
| g__Tumebacillus | 0.000732 | 0.003325 | 0.000421 | 0.003746 | 0.000842 | 0.002128 |
| g__Roseiarcus | 0.00133 | 0.000842 | 0.000998 | 0.000554 | 0.001064 | 0.000865 |
| g__Syntrophococcus | 0.000133 | 0 | 0 | 2.22E-05 | 0 | 0 |
| g__Cellulomonas | 0.000155 | 0.000177 | 4.43E-05 | 0.00031 | 2.22E-05 | 0.000222 |
| g__Coriobacteriaceae_UCG-002 | 0 | 0 | 0 | 2.22E-05 | 2.22E-05 | 0 |
| g__Anaerosalibacter | 4.43E-05 | 0 | 6.65E-05 | 4.43E-05 | 2.22E-05 | 2.22E-05 |
| g__Starkeya | 0 | 0 | 2.22E-05 | 0 | 0 | 0 |
| g__soil_bacterium_WF55 | 0.00031 | 2.22E-05 | 0.0002 | 0.000133 | 4.43E-05 | 6.65E-05 |
| g__Aeriscardovia | 4.43E-05 | 0 | 0 | 6.65E-05 | 0 | 0 |
| g__Enterorhabdus | 0 | 0 | 0.000133 | 6.65E-05 | 8.87E-05 | 0.000133 |
| g__Acetobacter | 0.000355 | 0 | 0 | 6.65E-05 | 6.65E-05 | 0 |
| g__Branchiibius | 0 | 0 | 2.22E-05 | 0 | 0 | 0 |
| g__Actinomycetospora | 8.87E-05 | 2.22E-05 | 6.65E-05 | 0 | 0 | 4.43E-05 |
| g__Eubacterium_brachy_group | 2.22E-05 | 0 | 0 | 0 | 0 | 0 |
| g__Ruminococcus_2 | 0.000266 | 0 | 0 | 0 | 0 | 0 |
| g__Frankia | 6.65E-05 | 0.000111 | 0.000177 | 8.87E-05 | 6.65E-05 | 4.43E-05 |
| g__Rubellimicrobium | 2.22E-05 | 0 | 0 | 4.43E-05 | 0 | 2.22E-05 |
| g__Thermincola | 4.43E-05 | 2.22E-05 | 0 | 0 | 0 | 0 |
| g__Ochromonas_sp._CCMP1393 | 2.22E-05 | 0 | 2.22E-05 | 0 | 0 | 2.22E-05 |
| g__Ideonella | 0.0002 | 0.000399 | 0.001685 | 0.000399 | 0.000266 | 0.000466 |
| g__Coelastrella_sp._M60 | 0.000111 | 2.22E-05 | 0.0002 | 2.22E-05 | 2.22E-05 | 0.000155 |
| g__bacterium_GLA1 | 0 | 2.22E-05 | 4.43E-05 | 0 | 0 | 0 |
| g__Kyrpidia | 2.22E-05 | 0 | 4.43E-05 | 8.87E-05 | 0 | 0 |
| g__Planctomyces | 0 | 0 | 0 | 0 | 0 | 6.65E-05 |
| g__Paucibacter | 6.65E-05 | 2.22E-05 | 0 | 0.000155 | 0.000177 | 0.000355 |
| g__Succinivibrionaceae_UCG-001 | 0 | 0 | 0 | 0 | 0.000909 | 0 |
| g__Aeribacillus | 2.22E-05 | 0 | 2.22E-05 | 2.22E-05 | 0 | 4.43E-05 |
| g__Actinocorallia | 2.22E-05 | 0 | 0 | 0 | 0 | 0 |
| g__Chlorotetraedron_incus | 0 | 2.22E-05 | 0 | 0 | 0 | 0 |
| g__Pseudenhygromyxa | 0 | 0 | 2.22E-05 | 0 | 0 | 2.22E-05 |
| g__Opitutus | 0.000975 | 0.001197 | 0.001596 | 0.001463 | 0.002594 | 0.00235 |
| g__Methylobacterium | 0.0002 | 0.0002 | 2.22E-05 | 0.000177 | 8.87E-05 | 6.65E-05 |
| g__Aquaspirillum | 0 | 0 | 0 | 0 | 0 | 2.22E-05 |
| g__Sphaerobacter | 0.000998 | 0.000599 | 0.000643 | 0.000865 | 0.000621 | 0.000443 |
| g__Demequina | 8.87E-05 | 0.000155 | 0.000133 | 0.0002 | 2.22E-05 | 0.000111 |
| g__Micavibrio | 0 | 6.65E-05 | 2.22E-05 | 0 | 2.22E-05 | 0 |
| g__Arenimonas | 0.000732 | 0.00082 | 0.000532 | 0.002372 | 0.001397 | 0.002217 |
| g__Candidatus_Arthromitus | 0 | 0 | 4.43E-05 | 0 | 2.22E-05 | 2.22E-05 |
| g__Pelagibacterium | 0 | 0 | 2.22E-05 | 2.22E-05 | 0 | 0 |
| g__Thermasporomyces | 0.000155 | 8.87E-05 | 0.000244 | 0.000488 | 0.000155 | 0.000244 |
| g__Ercella | 2.22E-05 | 0 | 0 | 8.87E-05 | 0 | 0 |
| g__Nitrolancea | 0.00102 | 0.000443 | 0.000842 | 0.00051 | 0.00031 | 0.000266 |
| g__Alicyclobacillus | 0.001197 | 0.000975 | 0.000909 | 0.001818 | 0.000887 | 0.000576 |
| g__Acidiphilium | 0.000554 | 0.000709 | 0.000665 | 0.000842 | 0.001064 | 0.000887 |
| g__Bryobacter | 0.014897 | 0.01452 | 0.010286 | 0.009998 | 0.01698 | 0.014254 |
| g__Chlamydiales_bacterium_CRIB_32 | 0 | 0 | 0 | 2.22E-05 | 0 | 4.43E-05 |
| g__Prevotella | 0 | 0 | 0 | 0.000709 | 0 | 0 |
| g__Chromobacterium | 0 | 0 | 6.65E-05 | 0 | 0 | 4.43E-05 |
| g__Campylobacter | 0 | 0 | 0 | 8.87E-05 | 0 | 0 |
| g__Turneriella | 2.22E-05 | 2.22E-05 | 0 | 8.87E-05 | 2.22E-05 | 0 |
| g__Kocuria | 0 | 0 | 0 | 0 | 4.43E-05 | 0 |
| g__Glutamicibacter | 2.22E-05 | 0 | 0 | 0 | 6.65E-05 | 0 |
| g__AKIW659 | 0.000177 | 0.000554 | 6.65E-05 | 0.000244 | 0.000399 | 0.000355 |
| g__Lysinibacillus | 2.22E-05 | 0.000133 | 4.43E-05 | 6.65E-05 | 4.43E-05 | 6.65E-05 |
| g__Aquabacterium | 0.000266 | 0.000155 | 0.005808 | 0.000266 | 0.000333 | 0.000421 |
| g__Anaerococcus | 0 | 0 | 0 | 0.000355 | 0 | 0 |
| g__Murdochiella | 0 | 0 | 0 | 2.22E-05 | 0 | 0 |
| g__Marmoricola | 6.65E-05 | 0 | 0 | 4.43E-05 | 2.22E-05 | 6.65E-05 |
| g__wastewater_metagenome | 2.22E-05 | 8.87E-05 | 0.000111 | 6.65E-05 | 8.87E-05 | 8.87E-05 |
| g__Terrimonas | 0.000288 | 0.000133 | 0.0002 | 0.000643 | 0.000288 | 0.000466 |
| g__Rhodoplanes | 0.000643 | 0.00051 | 0.000333 | 0.001264 | 0.000466 | 0.000798 |
| g__Anaeroplasma | 0 | 0 | 0 | 0 | 2.22E-05 | 0 |
| g__Bacillales_bacterium_Mi4 | 0.002483 | 6.65E-05 | 2.22E-05 | 2.22E-05 | 4.43E-05 | 0 |
| g__Microcoleus | 8.87E-05 | 0 | 2.22E-05 | 0.000111 | 0.00031 | 0.000288 |
| g__Azoarcus | 2.22E-05 | 0 | 0 | 8.87E-05 | 6.65E-05 | 0.000288 |
| g__Thermomonas | 0.000111 | 6.65E-05 | 4.43E-05 | 8.87E-05 | 0.000222 | 4.43E-05 |
| g__Elev-16S-1166 | 0 | 0 | 0 | 2.22E-05 | 0 | 2.22E-05 |
| g__H16 | 0.009776 | 0.019773 | 0.004278 | 0.005121 | 0.021636 | 0.016182 |
| g__Family_XIII_AD3011_group | 0 | 4.43E-05 | 8.87E-05 | 0 | 4.43E-05 | 0 |
| g__Clostridium_sensu_stricto_6 | 0 | 4.43E-05 | 0 | 0 | 8.87E-05 | 2.22E-05 |
| g__Eubacterium_coprostanoligenes_group | 0.000177 | 6.65E-05 | 0.000133 | 0.000222 | 0.000133 | 0.000111 |
| g__Clostridium_sensu_stricto_8 | 8.87E-05 | 8.87E-05 | 6.65E-05 | 4.43E-05 | 0.000111 | 8.87E-05 |
| g__Erysipelotrichaceae_UCG-006 | 2.22E-05 | 0 | 0 | 0 | 0 | 0 |
| g__Thermobacillus | 0.000355 | 0.000266 | 0.000244 | 0.000399 | 0.000155 | 0.000266 |
| g__Oscillibacter | 0 | 6.65E-05 | 0.000421 | 0.000333 | 8.87E-05 | 0.000133 |
| g__Lachnospiraceae_NK4A136_group | 0.000865 | 0.001729 | 0.003192 | 0.00286 | 0.00164 | 0.003392 |
| g__Bordetella | 0.001729 | 0.000599 | 0.000998 | 0.001308 | 6.65E-05 | 0.000111 |
| g__Rhodomicrobium | 0.000133 | 8.87E-05 | 0.000288 | 0.000111 | 0.000111 | 0.000266 |
| g__Citrobacter | 4.43E-05 | 2.22E-05 | 0.000842 | 0 | 0 | 0 |
| g__Roseburia | 4.43E-05 | 4.43E-05 | 0.000244 | 0.000155 | 0.000355 | 0.00031 |
| g__Microbispora | 0.001729 | 0.001153 | 0.001175 | 0.002815 | 0.001441 | 0.001153 |
| g__Lachnospiraceae_UCG-008 | 8.87E-05 | 4.43E-05 | 6.65E-05 | 0.000133 | 0 | 4.43E-05 |
| g__Eubacterium_fissicatena_group | 2.22E-05 | 0.000222 | 0.000333 | 2.22E-05 | 6.65E-05 | 0.000111 |
| g__Pseudarthrobacter | 0.000665 | 0.000155 | 8.87E-05 | 0.000266 | 0.000177 | 0.000133 |
| g__possible_genus_04 | 6.65E-05 | 0.000244 | 2.22E-05 | 0 | 0.000177 | 0.000133 |
| g__Candidatus_Solibacter | 0.015118 | 0.021436 | 0.004123 | 0.010973 | 0.025138 | 0.019663 |
| g__Kribbella | 0.000244 | 0.000222 | 0.000333 | 0.000399 | 0.000333 | 0.000554 |
| g__Actinospica | 0.002461 | 0.00399 | 0.012414 | 0.000953 | 0.001153 | 0.001773 |
| g__Thermoflavimicrobium | 2.22E-05 | 0 | 0 | 0 | 0 | 2.22E-05 |
| g__Sinomonas | 0.000333 | 0.0002 | 0.000222 | 0.000288 | 4.43E-05 | 0.000133 |
| g__Pseudoxanthomonas | 0.000244 | 0.000133 | 8.87E-05 | 4.43E-05 | 2.22E-05 | 0.000111 |
| g__Asticcacaulis | 0.000621 | 0.000532 | 0.000466 | 0.000488 | 0.000111 | 0.000222 |
| g__Schrenkiella_parvula | 2.22E-05 | 0 | 8.87E-05 | 2.22E-05 | 2.22E-05 | 6.65E-05 |
| g__Panacagrimonas | 0.000155 | 0.000111 | 8.87E-05 | 0.000111 | 0.000155 | 0.0002 |
| g__Filimonas | 0.000133 | 6.65E-05 | 8.87E-05 | 0.000133 | 6.65E-05 | 8.87E-05 |
| g__Thermoactinomyces | 0 | 4.43E-05 | 8.87E-05 | 6.65E-05 | 8.87E-05 | 4.43E-05 |
| g__Proteiniphilum | 6.65E-05 | 8.87E-05 | 8.87E-05 | 0.002483 | 8.87E-05 | 2.22E-05 |
| g__Romboutsia | 0.000155 | 0.000355 | 0.00133 | 0.000709 | 0.000155 | 0.000222 |
| g__Advenella | 8.87E-05 | 6.65E-05 | 2.22E-05 | 0.003259 | 2.22E-05 | 0.000288 |
| g__AKYG587 | 0 | 0 | 0 | 8.87E-05 | 2.22E-05 | 2.22E-05 |
| g__Pasteurella | 0.000111 | 0 | 0 | 0 | 0 | 0 |
| g__Pelotomaculum | 0 | 0 | 6.65E-05 | 0 | 2.22E-05 | 0 |
| g__Sneathia | 0 | 0 | 0 | 0.00031 | 0 | 0 |
| g__Actinophytocola | 2.22E-05 | 0 | 0 | 0 | 0 | 0 |
| g__SM1A02 | 0.000177 | 8.87E-05 | 0.00051 | 0.000443 | 0.000377 | 0.000599 |
| g__Sporosarcina | 0.000155 | 6.65E-05 | 0.000155 | 0.000133 | 0.000155 | 0.000244 |
| g__Candidatus_Amoebophilus | 4.43E-05 | 0 | 4.43E-05 | 2.22E-05 | 8.87E-05 | 4.43E-05 |
| g__Lactobacillus | 0.000532 | 0.0002 | 0.000909 | 0.001086 | 0.006251 | 0.000665 |
| g__Variibacter | 0.003192 | 0.004345 | 0.002704 | 0.004633 | 0.003702 | 0.004633 |
| g__Cohnella | 0.00031 | 0.000133 | 0.000133 | 6.65E-05 | 0.000177 | 8.87E-05 |
| g__Ferruginibacter | 6.65E-05 | 4.43E-05 | 2.22E-05 | 8.87E-05 | 0.000155 | 8.87E-05 |
| g__Luedemannella | 0.000643 | 0.000709 | 0.000488 | 0.000732 | 0.000975 | 0.001197 |
| g__Effusibacillus | 0 | 0 | 0 | 2.22E-05 | 0.000111 | 2.22E-05 |
| g__Parasutterella | 2.22E-05 | 2.22E-05 | 0.000111 | 0.000177 | 0.000133 | 8.87E-05 |
| g__Bradyrhizobium | 0.004434 | 0.004766 | 0.006562 | 0.006096 | 0.003525 | 0.005342 |
| g__uncultured | 0.000732 | 0.000222 | 0.000399 | 0.000687 | 0.000532 | 0.000621 |
| g__Aeromicrobium | 4.43E-05 | 8.87E-05 | 0 | 6.65E-05 | 8.87E-05 | 6.65E-05 |
| g__Ruminiclostridium_1 | 0 | 0 | 0.000111 | 0.000133 | 2.22E-05 | 0.000111 |
| g__Flavihumibacter | 2.22E-05 | 0 | 0 | 6.65E-05 | 2.22E-05 | 0.000111 |
| g__Chryseobacterium | 0.000643 | 0 | 0 | 2.22E-05 | 2.22E-05 | 0 |
| g__Erysipelatoclostridium | 8.87E-05 | 4.43E-05 | 0.0002 | 0.000111 | 0.0002 | 8.87E-05 |
| g__Clostridium_sensu_stricto_15 | 0 | 0 | 0 | 2.22E-05 | 0 | 0 |
| g__Leucobacter | 0 | 2.22E-05 | 2.22E-05 | 4.43E-05 | 2.22E-05 | 0 |
| g__Macellibacteroides | 2.22E-05 | 0 | 0 | 4.43E-05 | 2.22E-05 | 0 |
| g__Paludibaculum | 0 | 2.22E-05 | 2.22E-05 | 8.87E-05 | 2.22E-05 | 2.22E-05 |
| g__Telmatospirillum | 0.000222 | 0.000266 | 0.000155 | 0.000244 | 0.0002 | 0.000222 |
| g__Craurococcus | 0.000222 | 0.000266 | 0.000133 | 0.000687 | 0.000222 | 0.000399 |
| g__RB41 | 6.65E-05 | 8.87E-05 | 0.000155 | 0.000155 | 0.000244 | 0.000643 |
| g__Limnobacter | 0.000155 | 0.000133 | 0.000754 | 0.00031 | 8.87E-05 | 8.87E-05 |
| g__Filomicrobium | 0 | 0 | 0 | 0 | 0.000133 | 2.22E-05 |
| g__Thauera | 8.87E-05 | 0 | 0 | 4.43E-05 | 0 | 0 |
| g__Thermocrispum | 0 | 2.22E-05 | 0 | 0 | 2.22E-05 | 4.43E-05 |
| g__Gemella | 0 | 0 | 0 | 4.43E-05 | 0 | 0 |
| g__Pseudoscardovia | 0.000399 | 0 | 0 | 0 | 0 | 0 |
| g__Candidatus_Xenohaliotis | 0 | 0 | 0 | 0 | 2.22E-05 | 0 |
| g__Limnochorda | 0.000111 | 2.22E-05 | 4.43E-05 | 0.000177 | 0 | 4.43E-05 |
| g__Streptococcus | 4.43E-05 | 0 | 4.43E-05 | 2.22E-05 | 6.65E-05 | 4.43E-05 |
| g__Akkermansia | 2.22E-05 | 0.000266 | 0.000377 | 0.000709 | 0.000377 | 0.000842 |
| g__vadinBC27_wastewater-sludge_group | 0.000488 | 0 | 0.000222 | 0.001153 | 0.000177 | 0 |
| g__Legionella | 4.43E-05 | 0.000111 | 0.000155 | 0.0002 | 0.000133 | 0.000133 |
| g__Rhodopseudomonas | 0.000111 | 8.87E-05 | 0.000133 | 8.87E-05 | 4.43E-05 | 4.43E-05 |
| g__Sharpea | 4.43E-05 | 0 | 0 | 0 | 0 | 2.22E-05 |
| g__Nocardia | 0.000133 | 0.0002 | 0.000532 | 0.00031 | 4.43E-05 | 0.000443 |
| g__Thermobispora | 0.001973 | 0.002438 | 0.002527 | 0.004145 | 0.001862 | 0.00215 |
| g__Anaerolinea | 2.22E-05 | 2.22E-05 | 0 | 4.43E-05 | 2.22E-05 | 2.22E-05 |
| g__Virgisporangium | 2.22E-05 | 0.000355 | 0.000355 | 0.000355 | 0.000532 | 0.0002 |
| g__Isosphaera | 0.000554 | 0.000776 | 0.000887 | 0.000355 | 0.000643 | 0.000576 |
| g__Hirschia | 0.000333 | 0.0002 | 0.000355 | 0.000355 | 0.000333 | 0.000244 |
| g__Mycobacterium | 0.003081 | 0.00235 | 0.003347 | 0.002416 | 0.002305 | 0.002749 |
| g__Nocardiopsis | 0 | 2.22E-05 | 0 | 2.22E-05 | 0 | 0 |
| g__Candidatus_Omnitrophus | 0 | 0 | 0 | 0 | 4.43E-05 | 8.87E-05 |
| g__Sphingobacterium | 0.000266 | 0 | 0.001153 | 2.22E-05 | 6.65E-05 | 0 |
| g__Nevskia | 0.000399 | 0.000266 | 0.000421 | 0.000488 | 0.000909 | 0.000333 |
| g__Niastella | 0.000687 | 0.001374 | 0.000466 | 0.000288 | 0.000399 | 0.0002 |
| g__Tissierella | 4.43E-05 | 2.22E-05 | 4.43E-05 | 0.000266 | 0 | 0 |
| g__Terriglobus | 2.22E-05 | 0.000133 | 0 | 2.22E-05 | 2.22E-05 | 2.22E-05 |
| g__Actinoplanes | 0.003613 | 0.002483 | 0.005897 | 0.00736 | 0.002195 | 0.003791 |
| g__Sulfurifustis | 2.22E-05 | 0.000111 | 2.22E-05 | 0.000222 | 0.000443 | 0.000377 |
| g__Steroidobacter | 0.001663 | 0.00235 | 0.001884 | 0.002084 | 0.002283 | 0.001973 |
| g__Odoribacter | 0 | 2.22E-05 | 4.43E-05 | 0 | 4.43E-05 | 6.65E-05 |
| g__Andalucia_godoyi | 0 | 0 | 0 | 2.22E-05 | 0 | 0 |
| g__Sporocytophaga | 0.000333 | 8.87E-05 | 0.000288 | 2.22E-05 | 0.000133 | 8.87E-05 |
| g__Ruminiclostridium_9 | 6.65E-05 | 0.000222 | 0.000576 | 0.000443 | 0.000266 | 0.00031 |
| g__Ferrovibrio | 8.87E-05 | 2.22E-05 | 6.65E-05 | 4.43E-05 | 0.0002 | 0.000133 |
| g__Silvimonas | 6.65E-05 | 2.22E-05 | 0.000111 | 0 | 2.22E-05 | 0 |
| g__Paucimonas | 0.001264 | 0.000155 | 0.000177 | 0.000133 | 0.000111 | 6.65E-05 |
| g__Terrisporobacter | 2.22E-05 | 0 | 2.22E-05 | 0.000288 | 2.22E-05 | 0 |
| g__Candidatus_Nitrosoarchaeum_limnia_SFB1 | 0.000222 | 0.000244 | 0.000266 | 0.000377 | 0.000776 | 0.000599 |
| g__Alkanibacter | 0.000776 | 0.00031 | 0.000687 | 0.000333 | 0.000333 | 2.22E-05 |
| g__Uliginosibacterium | 4.43E-05 | 0 | 6.65E-05 | 2.22E-05 | 0 | 4.43E-05 |
| g__Parcubacteria_group_bacterium_GW2011_GWA1_60_11 | 0.000177 | 0.000133 | 0.000133 | 4.43E-05 | 0.00102 | 0.000599 |
| g__Labrys | 2.22E-05 | 6.65E-05 | 0.000111 | 0.000111 | 8.87E-05 | 6.65E-05 |
| g__Stenotrophobacter | 4.43E-05 | 2.22E-05 | 4.43E-05 | 0.000133 | 8.87E-05 | 8.87E-05 |
| g__Tistrella | 4.43E-05 | 2.22E-05 | 0.000133 | 0 | 0 | 0 |
| g__Altererythrobacter | 0.000288 | 0.000111 | 6.65E-05 | 0.000421 | 4.43E-05 | 0.0002 |
| g__Cytophaga | 0.000466 | 0.000244 | 0.000355 | 0.000177 | 0.0002 | 0.000288 |
| g__Bdellovibrio | 0.000887 | 0.000887 | 0.00031 | 0.000599 | 0.000576 | 0.000909 |
| g__Nocardioides | 0.005298 | 0.001973 | 0.002128 | 0.004877 | 0.001485 | 0.003635 |
| g__Porphyromonas | 0 | 0 | 0 | 0.000488 | 0 | 0 |
| g__Sphingobium | 0.000111 | 0.000177 | 2.22E-05 | 0.000111 | 6.65E-05 | 8.87E-05 |
| g__Chlorella_sp._CC-Bw-9 | 0.000244 | 8.87E-05 | 0.000554 | 0.000377 | 0.000266 | 0.000177 |
| g__Eubacterium_ventriosum_group | 0 | 0 | 4.43E-05 | 0 | 6.65E-05 | 0 |
| g__Ruminococcus_1 | 0 | 0 | 8.87E-05 | 8.87E-05 | 8.87E-05 | 6.65E-05 |
| g__Sedimentibacter | 4.43E-05 | 0 | 0.000133 | 0.000333 | 2.22E-05 | 0 |
| g__Candidatus_Alysiosphaera | 0.000133 | 0.000111 | 8.87E-05 | 0.000266 | 6.65E-05 | 0.000576 |
| g__Perlucidibaca | 0 | 0 | 6.65E-05 | 0 | 0 | 2.22E-05 |
| g__Conexibacter | 0.000643 | 0.000732 | 0.000244 | 0.002815 | 0.000355 | 0.000377 |
| g__Hahella | 4.43E-05 | 0 | 0.0002 | 0 | 0 | 0 |
| g__Dyadobacter | 0 | 4.43E-05 | 2.22E-05 | 0 | 0 | 0 |
| g__Geothrix | 0 | 0 | 0 | 4.43E-05 | 0.000133 | 0.000155 |
| g__Paeniclostridium | 2.22E-05 | 0 | 2.22E-05 | 8.87E-05 | 2.22E-05 | 2.22E-05 |
| g__Microbacterium | 0.000133 | 2.22E-05 | 2.22E-05 | 2.22E-05 | 0.000155 | 0.000222 |
| g__Solitalea | 0 | 0 | 0 | 0 | 0 | 8.87E-05 |
| g__Iamia | 0.000133 | 0.000155 | 0.000177 | 0.00031 | 0.000177 | 0.00051 |
| g__Atopobium | 0 | 0 | 0 | 2.22E-05 | 0 | 0 |
| g__Lysobacter | 2.22E-05 | 6.65E-05 | 6.65E-05 | 0.000177 | 0.000133 | 0.000222 |
| g__Bosea | 6.65E-05 | 2.22E-05 | 2.22E-05 | 0.000155 | 0.0002 | 0.0002 |
| g__bacterium_enrichment_culture_clone_auto112_4W | 4.43E-05 | 4.43E-05 | 6.65E-05 | 0 | 2.22E-05 | 2.22E-05 |
| g__Erysipelotrichaceae_UCG-002 | 0.000732 | 0 | 0 | 0 | 0 | 0 |
| g__Caenimonas | 2.22E-05 | 0.000155 | 2.22E-05 | 0.000155 | 0 | 6.65E-05 |
