# Supplementary material for: Effect of Pennisetum giganteum z.x.lin mixed nitrogen-fixing bacterial fertilizer on the growth, quality, soil fertility and bacterial community of pakchoi (Brassica chinensis L.)
Source: PLoS One. 2020 Feb 12;15(2):e0228709. doi: 10.1371/journal.pone.0228709 (PMC7015373; doi:10.1371/journal.pone.0228709)
Supplement: Attachment — Submitted filename: Response to Reviewers.docx [file pone.0228709.s002.docx]

**Reviewer #1:**

**Abstract**
L2-5, is it a sentence?

Yes, editor. It is a sentence. I use the *Pennisetum* giganteum z.x.lin mixed nitrogen-fixing biofertilizer. I test the growth, quality, physical and chemical properties of the soil and the bacterial community of the soil. I have revised this sentence in my paper. Please check it.
L21 most obviously ?

Dear editor, my most obviously is mean the content is the highest when compare with other groups. I had changed it into a comprehensible sentence. Please refer to it.
**Introduction**
L23: improve soil ? please modify it.

Dear editor, I deleted this sentence from the introduction section. Please refer to it.
**Discussion**
L19-20: presumably due to the application of chemical fertilizer will cause soil
acidification??? There are many mistakes like this. Please check you sentences carefully.

Dear editor. I made a revision to the discussion part, please refer to it.
What is the relationship between soil physical and chemical properties and yield of pakchoi (Brassica chinensis L.)? Please discuss it.

Dear editor, i have discuss the relationship between the physical and chemical properties of soil and the yield of Pakchoi according the requirements. Many studies have shown that fertilization changes the fertility and nutrient content of soil such as soil organic carbon and total nitrogen. It will directly drive the transformation of soil microbial community, increase or decrease the diversity of microbial community. Soil microorganisms can secrete growth active substances, such as auxin, cytokinin, zeatin, etc., to promote plant growth. Therefore, under the condition of fertilization, soil physical and chemical properties and microorganisms interact to jointly promote the growth of plants, and then improve the yield of plants.

How did you draw the conclusion that 25% chemical fertilizer reduction combined with Pennisetum sp. mixed nitrogen-fixing bacterial manure was the best practice to improve the growth and quality of pakchoi (Brassica chinensis L.)? To the extent, the conclusions section is a complete repetition of the main results. Please compare the difference of treatments and discuss it.

Dear editor, i have revised the conclusion part according to your requirements. In this study, as the ratio of chemical fertilizer and the *Pennisetum* *giganteum* mixed nitrogen-fixing bacterial manure is appropriate, it can promote the growth and quality of Pakchoi and improve the soil quality and the structure of soil bacterial community. When the reduction ratio of chemical fertilizer is large, it can also improve the quality of Pakchoi. However, it hinders the growth of Pakchoi due to the lack of nutrient supply. When the reduction ratio of chemical fertilizer is small, the nutrient supply is sufficient, but the change of soil environment leads to the reduction of beneficial bacteria in the soil, thus hindering the growth of Pakchoi and reducing its quality. Therefore, under the consideration of the factors such as the growth and quality of Pakchoi and its soil environment, the effect of 25% reduction of chemical fertilizer and the application of the *Pennisetum* sp. mixed nitrogen-fixing bacterial manure is the best. The results of this study can provide important theoretical basis for the rational reduction of chemical fertilizer and the scientific and reasonable application of the *Pennisetum* sp. mixed nitrogen-fixing bacterial manure. Please check it.

Please discuss how the soil properties affect the bacterial community structure and the growth of pakchoi (Brassica chinensis L.). Add latest references to enrich your paper.

Dear editor. In accordance with your requirements, I have added the latest references to the discussion part according to your requirements. The growth, activity and functional diversity of soil microbial community are affected by various soil physical and chemical properties, including soil pH, total nitrogen (TN), soil organic carbon (SOC) content and soil enzyme activity. Under the condition of fertilization, pH is the main driving factor to change microbial communities such as bacteria, fungi, archaea and protozoa. TN, SOC, enzyme activity and other factors are also important contributors to the composition of different microbial communities. Soil bacteria are very important for maintaining soil fertility and ecosystem function, and are usually sensitive to fertilizer input. Many studies have shown that fertilization changes the soil fertility status and the changes of nutrient content such as soil organic carbon and total nitrogen, will directly drive the transformation of soil microbial community, increase or reduce the diversity of microbial community. Some studies have also suggested that fertilization is indirectly used for soil microorganisms by changing the soil properties. Soil microorganisms can secrete growth active substances, such as auxin, cytokinin, zeatin, etc., to promote plant growth. Therefore, under the condition of fertilization, soil physical and chemical properties and microorganisms interact to jointly promote the growth of plants, and then improve the yield of plants. Please refer to it.

**Reviewer #2:**

**Title**
I cannot get the meaning of what the title is looking into, what is *Pennisetum* sp. Mixed what what. How do you mix a millet with N fixing bacteria to create a fertilizer? 我

Dear editor, i had revised the title of the paper to “Effect of *Pennisetum* giganteum z.x.lin mixed nitrogen-fixing bacterial fertilizer on the growth, quality, soil fertility and bacterial community of pakchoi (*Brassica* *chinensis* L.)”. I explained the preparation method in the material and method section. The activated *kosakonia radicincitans* and *bacillus mucilaginosus* were inoculated into LB liquid medium at a ratio of 1:1, oscillating culture 150-180 r/min under 30^o^C until logarithmic growth. 150-200 mL bacterial solution, 650-750 g of dry *Pennisetum* giganteum z.x.lin, 250-350 g of waste mushroom substrates of *Ganoderma lucidium*, and 100-150 mL nutrient solution were mixed into polyethylene film fermentation bag with breathing valve.
**Abstract**摘要
What is hm2, what unit is this?

hm2 is a hectare unit in China. I've changed it to the international unit (ha). Please refer to it
Line 5, including CK, what is this CK?

CK refers to control (non-fertilization). I have changed it to CK (non-fertilization) in the summary. Please refer to it.
The 1st sentence reading “to explore the effects… is not complete, what was done to explore these. At least give one background statement.

Dear editor, i have added a background note to the abstract and modified sentence as you requested. Biofertilizer plays a significant role in crop cultivation with reduced the inorganic fertilizer use.The effects of inorganic fertilizer reduction combined with *Pennisetum* giganteum z.x.lin mixed nitrogen-fixing biofertilizer on growth, quality, soil nutrients and diversity of soil bacterial community in the rhizosphere soil of pakchoi were studied.
Abstract is just too long, and the last sentence “the results of this study provide theoretical and scientific….what does this mean.

Dear editor, the abstract is too long so I deleted some sentences. Please check it. **Introduction**介绍
The section fails to justify why this study was undertaken, what other researchers have done and what this study will contribute. Its common knowledge that inorganic fertilizers are detrimental to the soil, so, what does this study seek to address, apart from presenting an alternative organic fertilizer.

Dear editor, i have modified the introduction according to your requirements. *Pennisetum* *giganteum* z.x.lin is belongs to Angiospermae, Monocotyledona, Gramineae and Pennisetum. It is a typical C4 plant, suitable for growing in tropical, subtropical and tempearate zone. *Pennissetum* *giganteum* z.x.lin has high nutritional value, good palatability and wide application. It can not only replace sawdust to cultivate edible and medicinal mushroom, solve the conflict of forest and mushroom industry development, but also be used as animal forage and has wide application in ecological management. Endophytic diaazotroph is a kind of microorganism that colonizes healthy plants and combines with host plants for nitrogen fixation. It plays an important role in nitrogen fixation, biological control and plant growth. Peng et al. found that the light and speed of rice leaves increased by 12% and the yield increased by 16% after inoculated with endophytic diaazotroph. Govindarajan et al. inoculated Burkholderia MG43 into sugarcane and found that it can replace half of nitrogen fertilizer and save 70 kg ha-1 nitrogen fertilizer. At present, there are many researchers on the endophytic nitrogen fixing bacteria of Gramineae and crops, but there are few reports on the endophytic nitrogen fixing bacteria of *Pennisetum giganteum* and its nitrogen fixing bacteria fertilizer. In order to provide theoretical basis for the scientific and reasonable application of *Pennisetum* giganteum z.x.lin mixed nitrogen-fixing biofertilizer and partial replacement of chemical fertilizer, the experiment was carried out to study its effect on the growth and quality of pakchoi, physical and chemical properties of soil and diversity of bacterial community.
Avoid using plurals of words, such as, Indicators (line 5); promotes (line 8); researches (line 18) among others.

Dear editor, I have changed it to indicator, promote and research according to your requirements. Please refer to it
Line 7, how does genetic diversity promote decomposition, this is not clear.

Dear editor, I'm sorry for the trouble caused by my English expression. I have revised this sentence into “It has the richest genetic diversity and can effectively promote the decomposition of organic matter and releases of nutrients”.
Line 11, chemical fertilizer, do you mean inorganic, rather use this term.

Dear editor, I have changed the chemical fertilizer to inorganic fertilizer according to your requirements.
Line 19, what is a microbial fertilizer, this is a new term.

Dear editor, what I want to express is biofertilizer. I have changed all of the microbial fertilizer to biofertilizer, and introduced its concept in the paper. Biofertilizers are products containing living cells of different types of microorganisms, which have an ability to convert nutritionally important elements from unavailable to available form through biological processes。
Line 29, small and large, meaning?

Dear editor, What I want to express is that there are many microorganisms in the soil. I'm sorry to have caused you trouble in reviewing your manuscript because of my own problems in English expression. For the revision, I deleted part of the content in the paper including this sentence.

Line 35, Pennisetum sp. Mixed… I can’t understand this part.

Dear editor, *Pennisetum* sp. mixed nitrogen-fixing bacterial fertilizer is refer to kind of biofertilizer，Because of the modification, some of the contents in the paper have been deleted, including this sentence.
Line 42, you need a clear Objective, it has to be SMART.

Dear editor, I have modified my objectives in accordance with your requirements. In order to provide theoretical basis for the scientific and reasonable application of *Pennisetum* giganteum z.x.lin mixed nitrogen-fixing biofertilizer and partial replacement of chemical fertilizer, the experiment was carried out to study its effect on the growth and quality of pakchoi, physical and chemical properties of soil and diversity of bacterial community.

Materials and methods材料与方法
This section is poorly written and does not allow the repetition of this study. Some of the sticking points are: which Pennisetum species, nutrient solution was brown sugar (meaning); how do u isolate bacteria from Pennisetum?; fermentation was end if the material became soft and dark (this is not a quantifiable character, how do you repeat this?).
250-350 g of mushroom waste, why mushroom, and which species.

Dear editor. I have modified this part according to your requirements. I use the *Pennisetum* *giganteum* z.x.lin species; the nutrient solution refers to the mixture of brown sugar, MgSO_4_ and calcium superphosphate, adding brown sugar as the carbon source, and combining certain calcium salt and magnesium salt to meet the nutritional needs of bacteria. I added the method of isolation of endogenous nitrogen fixing bacteria in the material preparation section. Put the sterilized samples of macromycorrhizal roots into a sterilized mortar, cut them with sterile scissors, and then add a proper amount of PBS buffer to grind them. Take 0.1 mL of the extract was coated on the Ashby solid medium plate, and the culture was inverted at 28~30^o^C for 2-3 days to observe the colony growth. Then, inoculate the single bacteria growing on the Ashby solid medium plate to the Nfb solid medium again for re-screening. Select the strains that can grow on the Nfb medium and the color of the medium changes from blue to green, and inoculate them on the Ashby solid medium again. Repeat in three times. Finally, inoculate the selected colonies on the Nfb slant medium and save them for future use and identification. *Bacillus mucilaginosus* was isolated from the rhizosphere soil of *Pennisetum* *giganteum* and preserved in the laboratory. After gradient dilution, the soil samples were coated on silicate bacteria culture medium and cultured in constant temperature incubator at 30^o^C for 48 h. According to the growth speed and morphology of the colony, the clear colony with mucilaginous protuberance is picked out from then separation plate, and then separated on the solid plate until the pure culture is obtained. 250-350g of waste mushroom substrates of *Ganoderma* *lucidium* cultivated with Juncao grasses was selected in this experiment, due to the use of microbial waste can be an utilization way of microbial waste and the growth of endophytic strains can be better satisfied. In addition, I added the quantitative detection method of fermentation end according to NY411-2000 <nitrogen fixing bacteria fertilizer> method.

Line 16, Material, use a better term.

Dear editor, I have changed it to preparation of test materials according to your requirements.
Line 17, 2 tested N fixing strains, give their full names, and how do you isolate these from a grass, Pennisetum.

Dear editor, I have given the full names of two strains according to your requirements and described the method of strain separation in the paper. *Bacillus mucilaginosus* was bought from local market. ***kosakonia radicincitans*** nitrogen fixation strain was isolated from *Pennisetum* giganteum z.x.lin and preserved in the laboratory. Put the sterilized samples of macromycorrhizal roots into a sterilized mortar, cut them with sterile scissors, and then add a proper amount of PBS buffer to grind them. Take 0.1 mL of the extract was coated on the Ashby solid medium plate, and the culture was inverted at 28~30^o^C for 2-3 days to observe the colony growth. Then, inoculate the single bacteria growing on the Ashby solid medium plate to the Nfb solid medium again for re-screening. Select the strains that can grow on the Nfb medium and the color of the medium changes from blue to green, and inoculate them on the Ashby solid medium again. Repeat in three times. Finally, inoculate the selected colonies on the Nfb slant medium and save them for future use and identification [28]. *Bacillus mucilaginosus* was isolated from the rhizosphere soil of *Pennisetum* *giganteum* and preserved in the laboratory. After gradient dilution, the soil samples were coated on silicate bacteria culture medium and cultured in constant temperature incubator at 30^o^C for 48 h. According to the growth speed and morphology of the colony, the clear colony with mucilaginous protuberance is picked out from then separation plate, and then separated on the solid plate until the pure culture is obtained

Line 18, preserved in the laboratory, not authors lab.

Dear editor, I have modified it as preserved in the laboratory according to your requirements.
Which buy from local market, correct gramma

Dear editor, I have modified it as “was brought from local market”.
Line 19-25; Chemical fertilizer….its not clear, I cant follow what was done here.

Dear editor, I'm sorry that my own English problems have caused you trouble in reviewing manuscript. I've revised this part in the paper. Pakchoi variety is No.2 *Brassica* *chinensis* from New Zealand was brought from local market; Inorganic fertilizer (N:P:K=18:6:6) was brought from Fujian AoLiGaoTa Fertilizer Co., Ltd; The stems and leaves of *Pennisetum* giganteum z.x.lin were selected from jointing stage or mature stage, crushed, dried at low temperature and passing through 100 mesh sieve. Waste mushroom substrate was choose Juncao grasses substrate only. The nutrient solution formula, brown sugar 30-50 g/L, MgSO_4_ 0.5-1.5 g/L, calcium superphosphate 10-20 g/L.
Line 31, how was the isolation done.

Dear editor. I have added the separation method to the paper as you requested. Put the sterilized samples of macromycorrhizal roots into a sterilized mortar, cut them with sterile scissors, and then add a proper amount of PBS buffer to grind them. Take 0.1 mL of the extract was coated on the Ashby solid medium plate, and the culture was inverted at 28~30^o^C for 2-3 days to observe the colony growth. Then, inoculate the single bacteria growing on the Ashby solid medium plate to the Nfb solid medium again for re-screening. Select the strains that can grow on the Nfb medium and the color of the medium changes from blue to green, and inoculate them on the Ashby solid medium again. Repeat in three times. Finally, inoculate the selected colonies on the Nfb slant medium and save them for future use and identification
What is hm2

Hm2 is a hectare unit in China, and I have changed it to the international unit ha.

Table 1m give the actual names of the species used.

Dear editor, I have given the actual name of the species used in Table 1 as you requested.

On soil chemical analysis, give the actual methods used to extract. Eg, available P was measured by colorimetry, how was the P extracted in the 1st place and provide references.

Dear editor, I have added the first step extraction method of organic matter, alkali hydrolyzed nitrogen, available phosphorus and available potassium in the paper according to your requirements

Data analysis, Factors with P < 0.05; what do you mean, which factors. Write this section clearly. Dear editor, what I want to say is to test indicators, not factors. I'm sorry that you are confused about my English. I've changed this sentence to “All statistical analyses were considered significant at the *P* ＜0.05 level”.

Morphology, what is this, I see nothing relating to plant morphology. A parameter like number of leaves is very useless to measure as this is a GxE interaction.

Dear editor, what I want to express is growth, not morphology. In addition, I have deleted the index of leaf number in my paper.

Avoid referring to your treatments as A, BC.

Dear editor, I have modified all processing names in the paper according to your requirements, and changed them to CK, DL, ZL, SL, FHF, JZ.

Table 2, +/- what value.

+/- refers to mean ± standard deviation，Results are presented as mean ± standard deviation (SD). I have explained it in the data analysis section

Table 3, that footnote is a repletion of stuff presented before.

Dear editor, I have deleted the repeated footnotes in the paper according to your requirements.

Discussion fails to link the results and explain what could have caused the responses. Avoid repeating results.

Dear editor, I had revised the discussion section as you requested and deleted some duplicate results. The growth, activity and functional diversity of soil microbial community were affected by various soil physical and chemical properties, including soil pH, total nitrogen (TN), soil organic carbon (SOC) and soil enzyme activity. Under the condition of fertilization, pH is the main driving factor to change microbial communities such as bacteria, fungi, archaea and protozoa. TN, SOC, enzyme activity and other factors are important contributors to the composition of different microbial communities. Soil bacteria are essential for maintaining soil fertility and ecosystem functions, and are often sensitive to fertilizer inputs. Many studies have shown that fertilization changes the soil fertility and changes the nutrient content such as organic carbon, total nitrogen etc. directly drives the transformation of soil microbial community and increases or decreases the diversity of microbial community. It is also believed that fertilization indirectly affects soil microorganism by changing soil properties. Soil microorganisms can secrete growth active substances such as auxin, cytokinin, zeatin, etc. to promote plant growth. Therefore, under the condition of fertilization, soil physical and chemical properties and microorganisms interact to jointly promote the growth of plant, and then improve the yield of plants

What is Naked oat leaves.

Dear editor, I want to express the meaning of “oat”. I have modified it in my paper.

**Conclusion**结论
Best effect, what effect are you referring to.

As the ratio of reduced fertilizer and combined *Pennisetum* giganteum z.x.lin mixed nitrogen-fixing biofertilizer is suitable, it can promote the growth and quality of pakchoi, soil quality and structure of soil bacterial community. When the reduction ratio of chemical fertilizer is large, it can also improve the quality of Pakchoi. However, it hinders the growth of Pakchoi due to the lack of nutrient supply. When the reduction ratio of chemical fertilizer is small, the nutrient supply is sufficient, but the change of soil environment leads to the reduction of beneficial bacteria in the soil, thus hindering the growth of Pakchoi and reducing its quality. Therefore, under the consideration of the factors such as the growth and quality of Pakchoi and its soil environment, the effect of 25% reduction of chemical fertilizer and the application of the *Pennisetum* sp. mixed nitrogen-fixing bacterial manure is the best. The results of this study can provide important theoretical basis for the rational reduction of chemical fertilizer and the scientific and reasonable application of the *Pennisetum* giganteum mixed nitrogen-fixing bacterial manure

Enhancing its quality, what is this, be specific to the parameter used to measure this.

Dear editor, enhancing its quality meaning nutritional value including soluble protein, soluble sugar, vitamin C and nitrate.
